# Development of *in vitro* methods to model the impact of vaginal lactobacilli on *Staphylococcus aureus* biofilm formation on menstrual cups as well as validation of recommended cleaning directions

**DOI:** 10.3389/frph.2023.1162746

**Published:** 2023-08-21

**Authors:** Maria Friberg, Kara Woeller, Vighter Iberi, Paolo Palacio Mancheno, James Riedeman, Lisa Bohman, Catherine C. Davis

**Affiliations:** ^1^ Baby, Feminine and Family Care Microbiology, The Procter & Gamble Company, Mason, OH, United States; ^2^ Baby, Feminine and Family Care, Global Product Stewardship, The Procter & Gamble Company, Cincinnati, OH, United States; ^3^ Corporate Functions Analytical, The Procter & Gamble Company, Mason, OH, United States; ^4^ Baby, Family and Feminine Care Analytical Chemistry, The Procter & Gamble Company, Cincinnati, OH, United States; ^5^ Data Modeling and Sciences, The Procter & Gamble Company, Mason, OH, United States; ^6^ Department of Medical Microbiology and Immunology, School of Medicine, Creighton University, Omaha, NE, United States

**Keywords:** menstrual cup, biofilm formation, staphylococcal toxic shock syndrome, safety, cleaning instructions

## Abstract

**Introduction:**

Menstrual cups (MC) are a reusable feminine hygiene product. A recent publication suggested that *Staphylococcus aureus* (*S. aureus*) biofilms can form on MCs which may lead to increased risk of menstrual Toxic Shock Syndrome (mTSS). Additionally, there is concern that buildup of residual menses may contribute to microbial growth and biofilm formation further increasing mTSS risk. Quantitative and qualitative analysis of *in vitro* tests were utilized to determine if *S. aureus* biofilm could form on MC in the presence of the keystone species *Lactobacillus* after 12 h of incubation. The methodology was based on a modification of an anaerobic *in vitro* method that harnesses the keystone species hypothesis by including a representative of vaginal lactic acid bacteria.

**Methods:**

MCs were incubated anaerobically for 12 h in Vaginal Defined Media (VDM) with the two morphologically distinct bacteria, *Lactobacillus gasseri* (*L. gasseri*) and *S. aureus*. Colony Forming Units (CFU) for each organism from the VDM broth and sonicated MC were estimated. In addition, a separate experiment was conducted where *S. aureus* was grown for 12 h in the absence *of L. gasseri*. Qualitative analysis for biofilm formation utilized micro-CT (µ-CT) and cryogenic scanning electron microscopy (Cryo-SEM).

**Results:**

Samples collected from the media control had expected growth of both organisms after 12 h of incubation. Samples collected from VDM broth were similar to media control at the end of the 12-h study. Total *S. aureus* cell density on MC following sonication/rinsing was minimal. Results when using a monoculture of *S. aureus* demonstrated that there was a significant growth of the organism in the media control and broth as well as the sonicated cups indicating that the presence of *L. gasseri* was important for controlling growth and adherence of *S. aureus*. Few rod-shaped bacteria (*L. gasseri)* and cocci (*S. aureus)* could be identified on the MCs when grown in a dual species culture inoculum and no biofilm was noted via µ-CT and cryo-SEM. Additionally, efforts to model and understand the validity of the current labeled recommendations for MC cleaning in-between uses are supported.

**Discussion:**

The data support continued safe use of the Tampax® cup when used and maintained as recommended.

## 1. Introduction

*Staphylococcus aureus* is a highly versatile and adaptable gram-positive pathogen. It can inhabit the skin and mucous membranes as a harmless commensal (1) and is most often found as a commensal vaginal organism in most reproductive-aged women (2, 3). With the insertion of *tst* in its genome*, S. aureus* can produce the superantigen, TSST-1. TSST-1 is associated with the pathogenesis of menstrual Toxic Shock Syndrome (mTSS); a rare, recognizable, and treatable disease that can occur in women who have low (<1:32) titers of neutralizing antibody to TSST-1 (4) during or around the time of menstruation (5). Although severe mTSS cases have occurred in women who have never used an intravaginal device, epidemiological research has linked intravaginal device use as a risk factor for mTSS (5). As a facultative anaerobe, *S. aureus* generates energy through aerobic respiration when present in an oxygen-rich environment but can switch to anaerobic respiration and fermentation for survival when present in oxygen deficient environments. While *S. aureus* can grow anaerobically, TSST-1 production requires oxidative metabolism. Certain strains of *S. aureus* can produce the superantigen, TSST-1 under the appropriate environmental conditions such as 21% O_2_ and 5% CO_2_ (6). Other environmental factors such as a neutral pH, are required for *in vitro* TSST-1 production (7, 8).

Several physiological mechanisms maintain the health of the vagina including the composition (richness and diversity) of the vaginal microbiome. Maintenance of high numbers of vaginal lactic acid bacteria have been identified as the hallmark of health. Since 1896, emphasis has been on members of the genus *Lactobacillus* as a keystone species due to their ability to ferment sugars to produce lactic acid (9–11). Lactic acid bacteria that predominate in the vagina of reproductive-age women (12) metabolize extracellular glycogen stored in epithelial cells into lactic acid by anaerobic glycolysis and fermentation—thereby lowering the vaginal pH to create an inhospitable environment for many pathogenic bacterial and viral species (13). Importantly, lactic acid bacteria are thought to serve as the keystone species in maintaining the health of the vagina (14–16) and have been shown to prevent the overgrowth of opportunistic pathogens such as *S. aureus* (14, 17) which are also found in vagina of some women (2). Lactic acid bacteria, found along the vaginal walls and cervix in reproductive age women (18) have been shown to suppress TSST-1 production in some populations of women; additional work is needed to define the characteristics responsible for this suppression of TSST-1 (19). Different species of lactobacilli can produce a variety of compounds such as lactic acid (*L. gasseri)* (20), hydrogen peroxide (e.g., *L acidophilus, L. crispatus*, *L. gasseri*) (21) and even tetramic acids (*L. reuteri*) (22) which have been shown to reduce IL-8 in vaginal epithelial cells (23) and even inhibit quorum sensing activities of *S. aureus* (24).

In nature, microorganisms, including *S. aureus,* exist primarily by attaching to and growing upon abiotic and biotic surfaces (25, 26). These surface-attached microbial agglomerations were named “biofilms” for the first time in 1978 (27). Contributing to the potential pathogenicity of *S. aureus* is its ability to form biofilms where single cells are aggregated and embedded in a protective matrix that may or may not be adhered to a surface (25). Once in this aggregate, cell-signaling may occur through quorum sensing via the *agr* system (28). *Agr* has various biological functions: (1) regulating the expression of staphylococcal virulence factors and (2) facilitating the structuring and detachment of bacteria biofilms. These functions are crucial for the pathogenesis of staphylococci and are always associated with the pathogenicity of highly virulent *S. aureus* (29). This system controls a variety of virulence mechanisms including production of TSST-1.

Biofilms have been identified on human tissue surfaces, including human vaginal epithelial cells (30–32). However, these adherent bacteria do not typically mature into thick biofilms given that there is a complete turnover rate of 96 h by the vaginal tissues (33). Adherent bacteria and biofilms that originate from the vaginal microbiota have been identified on a variety of removable vaginal medical devices such as tampons, pessaries, and menstrual cups [MC(s)] (31, 32) with the biofilm thickness and diversity related to the labeled wear time (34). In these cases, active disease is absent in a high percentage of women (31, 32). Because TSST-1 positive strains can produce higher concentrations of TSST-1 when cultured in biofilm compared to individual, adherent bacteria (6), it is an important element of the overall safety program to understand the potential for biofilm to form on intravaginal durable medical devices.

A MC is a type of reusable feminine hygiene product that is a small, flexible funnel-shaped device that many women use as an eco-friendly alternative to tampons and has been commercially available in the US for managing menstrual flow since 1937 (35). The MC is folded and inserted into the vagina below the cervix, where it collects rather than absorbs menstrual flow. Depending on the manufacturer and the size of the cup, MCs can hold 10–38 ml of menses. MCs can be worn up to 12 h, depending on a women's menstrual flow, then removed and menses disposed. As durable products, MCs are cleaned in between use instead of being disposed after a single use (36). MCs are often made of medical-grade silicone, while a few brands use thermoplastic elastomer (TPA) or rubber (37).

The Tampax MC, which is the focus of this research, is comprised of a single material, liquid silicone rubber, has a recommended use period of one year. Recommended cleaning instructions (as indicated on the product insert) include washing the cup with soap and water between uses throughout the menstrual cycle and boiling the cup for five to seven minutes prior to the initial use and at the end of each menstrual cycle, prior to storing the cup (38).

## 2. Research questions

A recent publication by Nonfoux et al. (39) using *in vitro* testing methods stated that *S. aureus* biofilm formation on menstrual cups after 8 h can lead to bacterial colonization on the product that potentially alters mTSS risk and the safe use of MCs. The same publication suggested that current cleaning protocols are not sufficient to remove biofilm (39). Subsequently, a publication by Wunsch et al. (40), investigated various cleaning techniques noting that cleaning with cold tap water (with or without soap) followed by steeping in boiled water for 5 min was most effective at reducing viable bacteria. Thus, the objectives of this research report centered on whether *S. aureus* biofilms would form on the MC in the presence of lactic acid bacteria (keystone species) and if the instructions on the current package of the Tampax MC were sufficient manage the cleaning (both effectively and easily) of the MC in between uses. The specific research questions included:

1. What are the best environmental conditions to use in an *in vitro* test system to mimic the exposure of the menstrual cup to the vaginal environment and microbiota?
2. Does the presence of *L. gasseri,* a vaginal lactic acid bacteria (acting as keystone species) alter the likelihood of *S. aureus* biofilm formation quantitatively or qualitatively on the MC when the two organisms (dual species system) are incubated for 12 h (labeled wear time of the Tampax MC) in cell densities found in the human vagina?
3. Are the current cleaning instructions for the Tampax cup sufficient to remove adherent bacteria based on the results of *in vitro* test analyses?

## 3. Materials and methods

### 3.1. Test articles (MC)

The Tampax**®** MC (The Procter & Gamble Company, Cincinnati, OH) is composed of Class VI medical grade silicone which meets the highest Class VI medical grade classification following the *U.S. Pharmacopeia criteria for the classification of plastics* (41). Three different product iterations of the Tampax MC were used in this research: The currently marketed Tampax menstrual cup is comprised of QP1–40 Class VI medical grade silicone as supplied by Dow/Dupont. The QP1–20 menstrual cup is not marketed but is identical to the QP1–40 menstrual cup except for minor differences in starting materials. Two different prototypes of the QP1–20 cup were tested; the only difference between these two MCs (noted as Prototype 1 and Protype 2) is the initiation of a 4 h curing step which was utilized for Prototype 1 only. Tampax MCs come in “regular” and “heavy” flow sizes; all testing was conducted on the “heavy flow” size of the Tampax MC as it is the cup with greatest surface area, and therefore, regarded as the worst case scenario for biofilm formation. The MCs used in both clinical and *in vitro* experiments as outlined in this paper are also summarized in Supplementary Table S–1.

### 3.2. Bacterial strains

All strains were selected from the ATCC strain collection (https://www.atcc.org/) and selected because they were sourced from the human vagina. *S. aureus subsp aureus* Rosenbach ATCC 33589 D1470 (587), a facultative anaerobe isolated from vagina of TSS patient and *L. gasseri* ATCC 9857, an aerotolerant anaerobe isolated from the vagina, were used throughout the quantitative and qualitative biofilm formation assays. *S. aureus Rosenbach* ATCC 33589, and *Escherichia coli* ATCC 8739, were used in the MC cleaning study. *S. aureus,* a Gram-positive organism, was chosen because it was the relevant organism for this study. *E. coli*, a Gram-negative enteric bacterium, was chosen as a second organism because it is traditionally used in cleaning, disinfection, and sterilization studies to measure log reduction and sufficiency of the applied cleaning methodology (42–44).

### 3.3. Media

Chemically Defined Medium (CDM), originally described by Geshnizgani (45) and referred herein as Vaginal Defined Media (VDM) is a complex formula, compositionally similar to that of the female genital tract of reproductive women and contains hemin and mucins that provide a nutrient rich environment that mimics the *in vivo* ecosystem. This formula is sufficient to support long term growth of microorganisms in safety studies that span a duration greater than several hours and is most appropriate for quantifying bacteria growth and/or biofilm formation in the presence of vaginal medical devices as indicated by several published studies (38, 46–48). Formula refinements were reported by Elkins et al. (49), for use in a dual-species *in vitro* system and it is this medium, formulated at a pH of 6.5 ± 0.2 to simulate the near neutral pH of the vagina environment during menstruation that was used in the quantitative and qualitative biofilm studies.

Artificial Menstrual Fluid (AMF) is a simple and thicker suspension media commonly used to investigate fluid handling properties of absorbent menstrual products. AMF was selected for the cleaning experiments due to its viscosity which allowed better adherence of the artificial microbial soil to the cup.

### 3.4. Experimental procedures

#### 3.4.1. Research question #1: determination of appropriate *in vitro* environmental conditions

Two independent experiments were conducted to determine the best environmental conditions, aerobic or anaerobic, to use in an *in vitro* test system to mimic the exposure of the menstrual cup to the vaginal environment and microbiota. The first experiment aimed at understanding if the menstrual cup could introduce oxygen during MC insertion potentially altering the normally anaerobic nature of the vagina; the second experiment aimed at understanding if the position of the menstrual cup would alter the physical opening of the vagina allowing entrance of oxygen during MC wear.

##### 3.4.1.1. Understanding potential to introduce oxygen with Tampax menstrual cup insertion

**Figure 1:**
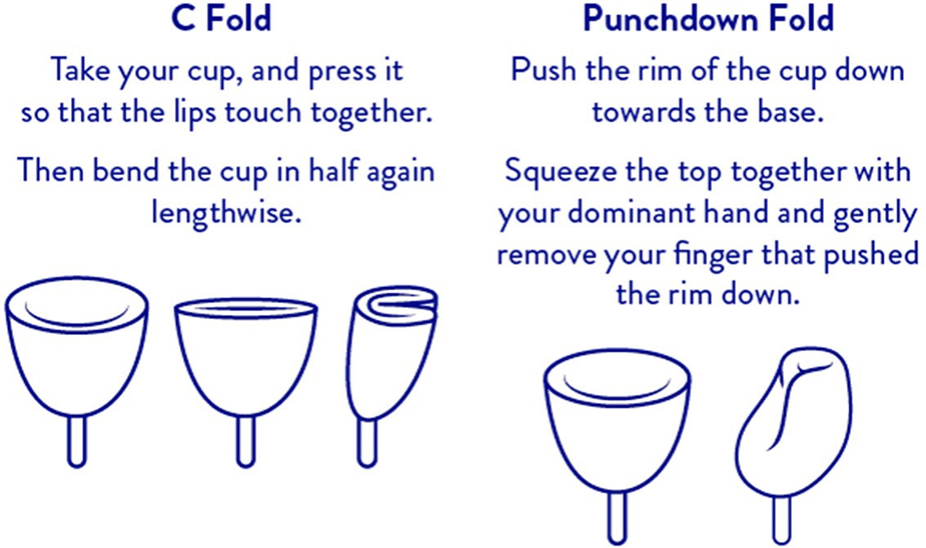
Label instruction for Tampax menstrual cup insertion via two different methods.

The following procedure was used to determine possible residual oxygen remaining in the Tampax cup after folding and insertion into the vagina and then correlating the measurement with oxygen content based on ideal gas law calculations. Two folding techniques described in the package insert of the Tampax cup are: the C fold and punchdown fold (Figure 1). A heavy flow Tampax menstrual cup was held upright (cup open end pointed upward and level to avoid spilling) and filled to the rim using Millipore water. The cup was folded either using the C fold or punchdown fold technique causing some of the water to be expelled leaving water only in the void volume (volume remaining after folding). Water remaining in the cup was measured on an analytical balance. The ideal gas law, (PV=nRT) was used to calculate the amount of oxygen that would be present in the cup after folding where:

P** =** 1 atm.
V = void volume in cup (in liters)
n = moles of air
R = gas constant (0.08206 L·atm/K·mol)
T = 310.15 K (body temperature in Kelvin).

The equation was solved for *n* (number of moles of air) and multiplied by the concentration of oxygen in the atmosphere (20.95%).

Subsequently, the time to reach low or zero O_2_ was calculated using the starting (131.00 torr) and ending (98.00 torr) partial pressure from Wagner et al. (50) and the following assumptions to calculate the rate of change of O_2_: pressure remains constant [total pressure at elevated body temperature calculated using Gay Lussac's Law P_1_/T_1_ and P_2_/T_2_ where P_1_ is pressure of air at ambient temperature (1 atmosphere), T_1_ = 273.15 K, P_2_ is unknown and T_2_ = 310.15 K (body temperature); solving for the unknown (P_2_ = the pressure of the void volume at body temperature) yields 1.13 atm or 862.95 torr at 37°C], the decrease in partial pressure of O_2_ increases partial pressure of all other gasses in air, an increase in partial pressure of N_2_ and other gasses in air is proportional to volume decreases via $P_{1}V_{1}=P_{2}V_{2}$ and the volume decrease is due to absorption of O_2_ only.

As Robb (51) noted, oxygen can diffuse through silicone rubber, 0.014 cm^3^/min, but the diffusion rate per cm^2^ is quite slow relative to the rate of absorption by the body (50). A silicone rubber Tampax MC ranges in thickness from approximately 3 to 5 mm, a fairly thick barrier to O_2_ on the timescale of this study.

The mechanism of O_2_ decline is suspected to be by absorption through capillaries and subsequently into the tissues/cells that are supplied by local capillaries (52, 53). Finally, movement of the diffused oxygen will enter epithelial cells which is crucial for mitochondrial respiration (54, 55) Also, bodily fluids, such as menses, which flow from the uterus into the vaginal vault via the cervix are moving and in flux (56). It is postulated that the small amount of O_2_ in the cup has dissipated into the surrounding tissues within 30 min.

##### 3.4.1.2. Clinical study to determine anatomical location of menstrual cup after insertion

An observational study was designed (Sponsor: The Procter & Gamble Company), and the protocol and informed consent documents sent to Advarra IRB (Columbia, MD, USA) for review. The study was conducted in compliance with the applicable Federal Regulations Guidelines for Good Clinical Practice (57). Inclusion and exclusion criteria were reviewed with each potential subject and if all inclusion and none of the exclusion criteria were met, the subject was accepted into the study after completing informed consent. Inclusion and exclusion criteria are contained in Supplementary Table S–2. Effort was made to enroll a group of women with a wide range of Body Mass Indices (BMIs) representative of the user population; BMI of the premenopausal women was recorded. Subjects had regular periods, had at least 1 child and were comfortable using intravaginal devices. MRI scans were obtained at Proscan Tri-County, OH. No imaging aids were added to the MC except an imaging marker was placed at the vaginal opening. Images were taken: scout (obtained for localization purposes allowing the technician to select the area of dedicated image acquisition), supine, axial, sagittal, coronal, and sagittal (during valsalva maneuvers). Images were analyzed using RadiAnt DICOM viewer.

#### 3.4.2. Research question #2: does the presence of *L. gasseri* alter the likelihood of *S. aureus* biofilm formation quantitatively or qualitatively?

In this study, the determination of biofilm was based on characterization of biofilm viability using quantitative indirect methods (cfu/ml determination following sonication) complemented by direct qualitative measure to visualize the morphology (μ-CT and cryo-SEM).

##### 3.4.2.1. Quantitative evaluation of biofilm formation

**Figure 2:**
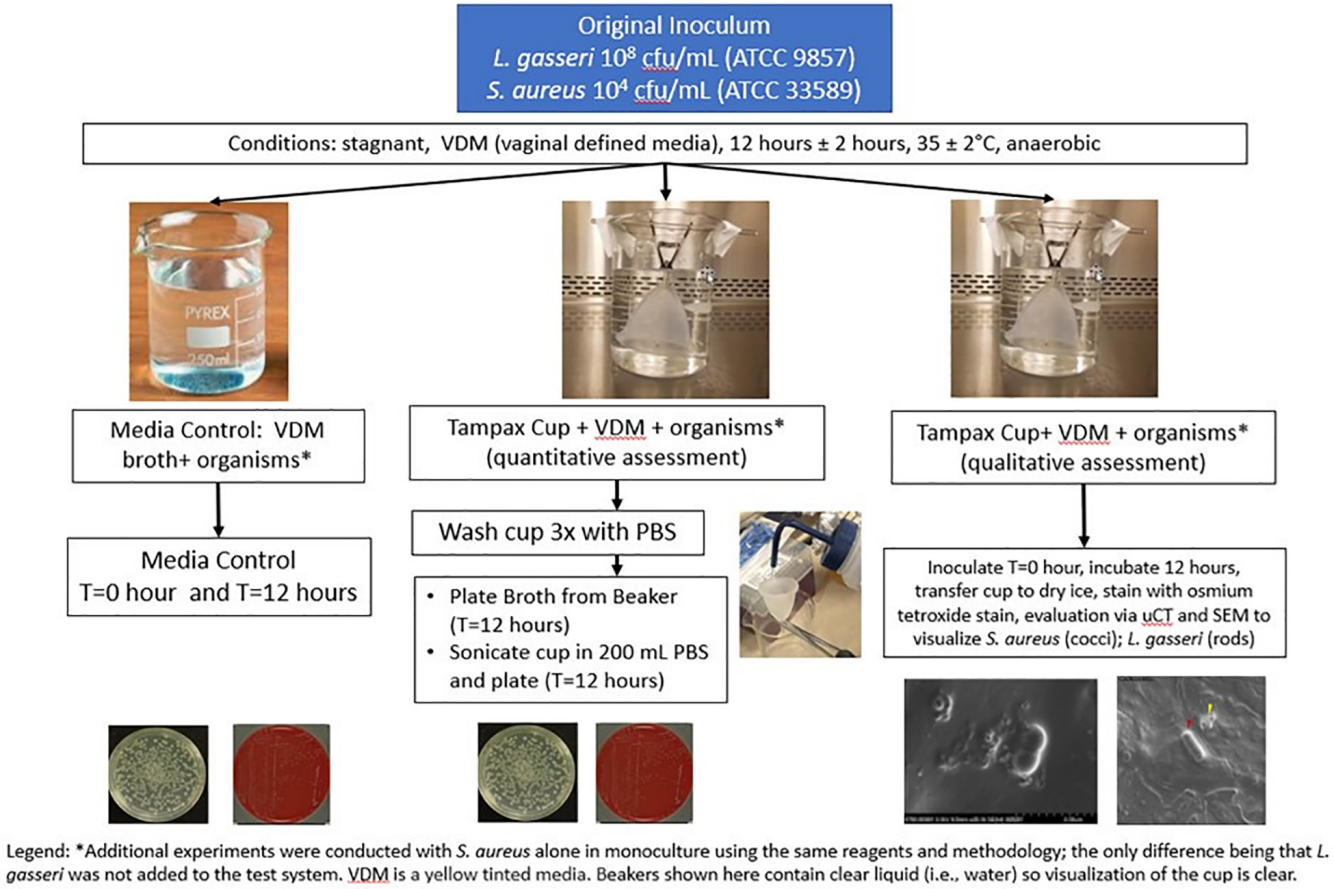
Methodology for biofilm formation study—quantitative and qualitative assessment overview.

The quantitative biofilm formation study was performed multiple times. Each experiment included one beaker designated as the media control (Vaginal Defined Media, VDM, and a dual species culture of *S. aureus* and *L. gasseri*) and at least 3 beakers designated as broth (VDM, a co-culture of *S. aureus* and *L. gasseri* and the Tampax cup). In experiments which encompassed a qualitative assessment of biofilm formation, an additional beaker was included so that the total number of beakers for quantitative assessment remained at 3 (Figure 2).

Each cup was submerged and suspended upside down in the vertical position inside a sterile beaker containing 250 mL of VDM with a dual species culture of 10^4^ cfu/mL *S. aureus* (ATCC 33589) and 10^8^ cfu/mL *L. gasseri* (ATCC 9857). The original *S. aureus* cell density was selected based on human vaginal washing samples from Johnson, et al. (58), and *L. gasseri* cell density was selected based on analysis of microbial composition of human vaginal swabs (59). Based on the results of earlier experiments (section 3.4.1.1 and 3.4.1.2) supporting anaerobic conditions for the *in vitro* study, the beakers were incubated in an anaerobic chamber (Anaerobe Chamber Coy Model Type B Serial number AC12020) at 35°C ± 2°C without shaking for 12 h. Following this 12 h period, samples were collected for plating and enumeration from the dual species culture media control and broth using selective media under appropriate growth conditions: Mannitol Salt Agar (MSA) for *S. aureus* (aerobic) and Human Bilayer Tween (HBT) (Remel R0147/VWR 90008-708) for *L. gasseri* (anaerobic). Plates were incubated for 48 h ± 2 h at 35 ± 2°C and cfu/mL were determined separately for both *S. aureus* and *L. gasseri.* The maximum incubation time of 12 h was selected since the labeled wear time for the Tampax MC is up to 12 h.

Additionally, the MC was removed from the broth and gently rinsed 3 times with 300 mL phosphate-buffered saline (PBS) (Difco), before sonicating in 200 ml of PBS for 1 min using the BactoSonic instrument (Bandelin Electronics). Sonication was utilized to release any adhered organisms from the menstrual cup, samples were taken from the PBS for plating and enumeration and cell density counts for both *S. aureus* and *L. gasseri* were determined as described previously for the media control and broth samples. The simple ultrasound sonication protocol applied prior to cfu determination has been demonstrated to disrupt large planktonic aggregates of *S. aureus* otherwise recalcitrant to disruption by shear forces to obtain accurate estimates of cell numbers in laboratory liquid cultures of *S. aureus* (60).

From these data points, the following comparisons were made:

• Cell density values (cfu/mL) obtained from three cup types made of medical grade silicone were compared to understand if there were significant differences in organism recovery between the cups at 12 h.
• The number of organisms recovered from the media control (without MC) was compared to the number of organisms recovered from the broth (with MC) to evaluate the impact of the cup on organism growth.
• The number of non-adherent (planktonic) organisms recovered from the broth was compared to the organisms recovered from the sonicated cup to quantify the number of organisms adhered to the cup.

In all comparisons, cell density data were log transformed prior to statistical analysis. Additionally, each data point recovered from the sonicated cups was normalized against the broth volume before used for statistical analysis to allow for appropriate comparison (200 mL PBS vs. 250 mL media).

Across all experiments, the total number of datapoints for Media Control and Broth was *n* = 5 and *n* = 16, respectively. Each of these 16 Broth beakers contained a single menstrual cup. Two of these cups, randomly selected, were removed from the Broth, and sent without further manipulation (e.g., no rinsing/sonication) for qualitative assessment of *S. aureus* and *L.* gasseri via µ-CT and cryo-SEM (Section 3.4.2.2.1). Cell density values were not determined from the cups sent for qualitative analysis. The remaining 14 cups were processed for quantitative biofilm evaluation after sonication. Fourteen successful data points were generated for *L. gasseri,* and eleven successful data points were generated for *S. aureus.* Three *S. aureus* data sets were excluded due to being outside of countable range.

An additional experiment was conducted with *S. aureus* alone in monoculture (*n* = 3) using the same reagents and preparation; the only difference being that *L. gasseri* was not added to the test system. In this study, both quantitative and qualitative assessment was conducted; one MC, randomly selected, was submitted for µCT and cryo-SEM analysis to identify any possible biofilm formation.

##### 3.4.2.2. Qualitative evaluation of biofilm formation

Qualitative analyses via μ-CT and cryo-SEM were conducted for MCs that were incubated in the presence of the microbial dual species culture of *L. gasseri* and *S. aureus* as well as monoculture of *S. aureus*. Consistent with the published literature (31), biofilm was defined as ≥ 7 bacterial cells clustered to form a surface-attached structure.

###### 3.4.2.2.1. Contrast-enhanced micro-computed tomography: biofilm tracking of menstrual cup

MCs were incubated with the dual species of cultured bacteria (*L. gasseri* and *S. aureus)* for 12 h as described above in the quantitative biofilm formation study. At the end of 12 h of incubation, MCs were removed from the broth and, without rinsing or sonication, immediately placed on dry ice to preserve the morphology of adherent bacteria. MCs were transported to the µ-CT imaging facility where they were kept at −80°C until staining.

Menstrual cups were thawed to ambient temperature for 10 min. Subsequently, cups were exposed to osmium tetroxide (OsO_4_) vapor for 2 h at 25 °C. Osmium exposure was completed inside an airtight container inside a chemical hood. The proposed underlying staining mechanism and the interaction of OsO_4_ with bacteria is an active process of oxidation, which refers to the binding of osmium metal species onto molecules of unsaturated lipids, and other carbohydrates present in the cell membranes via oxidation (61, 62).

3D x-Ray images were obtained on a µ-CT instrument, Scanco µCT 100HE (Scanco Medical AG, Switzerland). The µ-CT instrument used was a shielded cone beam microtomograph with an x-ray tube with a micro-source and an adjustable focal spot diameter. The x-ray beam passes through the stained menstrual cup, where x-rays are attenuated based on the specimen's osmium accumulation. Hence, the extent of attenuation correlates to the mass density the x-rays pass through. The attenuated transmitted x-rays continue to a digital detector array and generate a 2D projection of the sample. A 3D image of the cup was generated by collecting hundreds of individual 2D projections at different directional angles as the sample was rotated (63). The MC was placed inside a 73 mm low density holder and kept at ambient temperature and relative humidity. The system operated with the following settings: energy level of 45 kVp; 3,000 projections; 55 mm field of view, FOV; and an averaging of 5. Hence, the µ-CT x-ray instrument could acquire a dataset with a high isotropic spatial resolution of ∼20 μm per pixel.

After scanning and subsequent completion of data reconstruction, data was transformed with the AVIZO (Thermofisher, V2020) software for image analysis. Maximum projections were created to identify areas of high osmium concentration and appeared as bright spots on the 3D image. These high osmium accumulation areas were marked and served as a guide during cryo-SEM imaging.

###### 3.4.2.2.2. Cyro-SEM: biofilm tracking of menstrual cup

An area of high contrast within an osmium-stained menstrual cup was excised and securely mounted on a holder with an equal mixture of OCT compound and colloidal graphite. The mounted sample was flash-frozen in liquid nitrogen slush and transferred under vacuum into a *Quorum PP3010T* cryo-prep chamber. Additional sublimation was performed on the sample at −85°C for 10 min and sputter-coating with a layer of Pt for 100 s to mitigate sample charging. Scanning electron microscopy (SEM) imaging was performed using a *Hitachi Ethos NX5000.* The use of cryo-SEM is recognized as an invaluable tool in the visualization of biofilm that can reveal details about biofilm structure and topography that other microscopy techniques cannot (64, 65). This tool, while limited in use due to the expense of equipment and a skill set necessary to operate the equipment and interpret the images, avoids the complication associated with complex chemistry-driven methodologies (e.g., microscopy such as FISH probes) that require oxygen to work rendering them inconducive to visualization of *L. gasseri* which requires anaerobic environmental conditions.

#### 3.4.3. Research question #3: Are the current cleaning instructions for the Tampax cup sufficient in removal of adherent bacteria?

**Figure 3:**
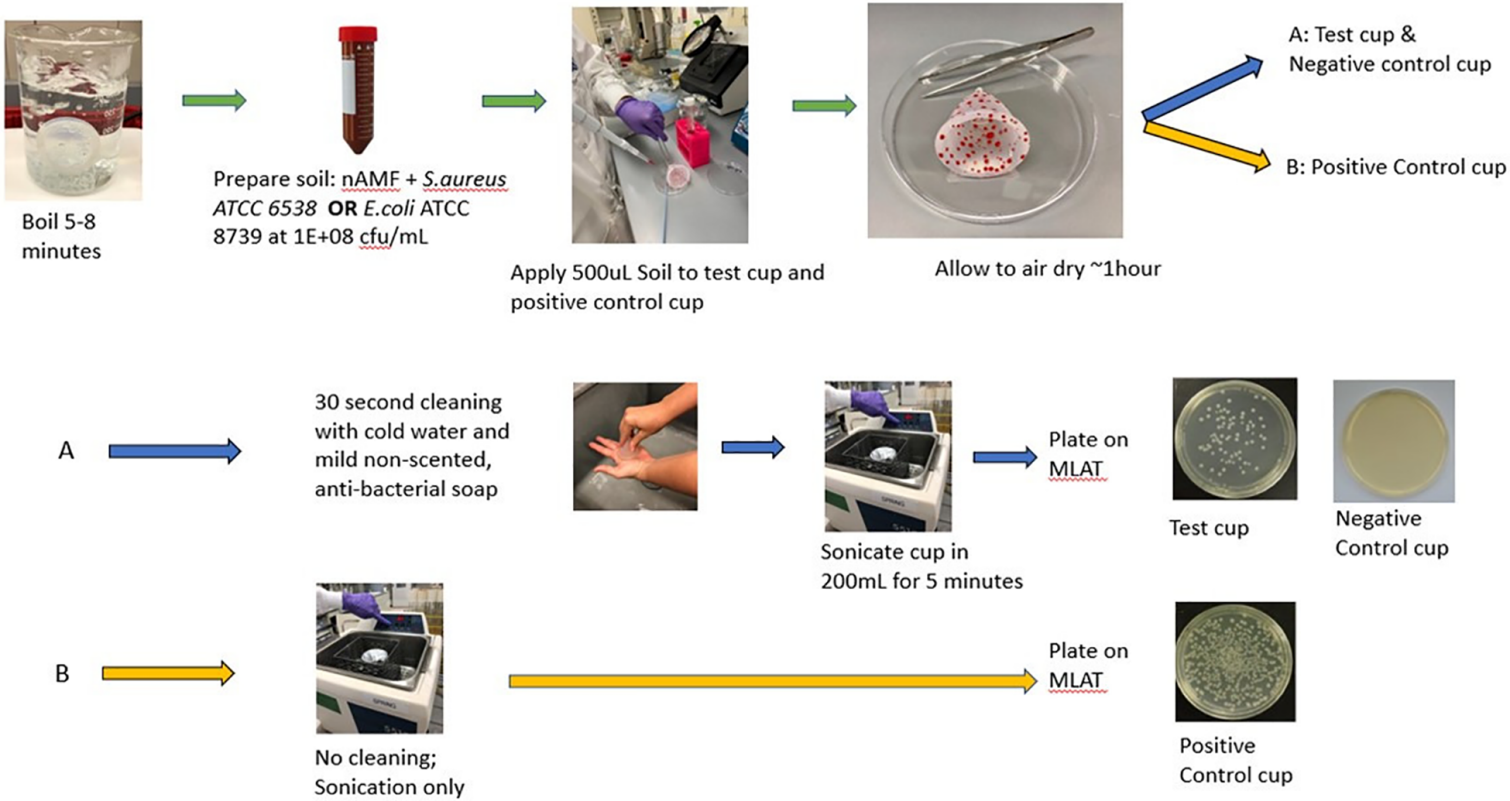
Methodology for the 30 seconds menstrual cup cleaning study.

This experiment consisted of a test cup [artificial soil (as defined below) with cleaning], a positive control cup (artificial soil without cleaning) and a negative control cup (no artificial soil, with cleaning). Prior to the start of the experiment, all MCs were boiled for 5–8 min to ensure a sterile device, consistent with packing insert directions. Artificial soil (e.g., organic matter) was prepared by combining 3 mL of artificial menstrual fluid (AMF) (Q-Labs Cincinnati, OH) and 3 mL of an inoculum of either *E. coli* (ATCC 8739) or *S. aureus* (ATCC 33589) in saline. A starting inoculum of 1.0 × 10^8 ^cfu/ml was chosen for both *S. aureus* and *E. coli* to enable evaluation of a sufficient log reduction consistent with available cleaning standards (e.g., FDA). AMF comprised of sterile sheep blood and other proprietary materials was prepared a maximum of 24 h in advance of each experiment; organisms were prepared immediately prior to each experiment. The AMF/organism mixture was vortexed for 1 min and then 500 µl of the mixture was spread via sterile loop on the internal and external surfaces of the test cup and positive control cup and allowed to dry at ambient temperature for approximately 1 h. After sufficient drying, the test cup and negative control cup were cleaned externally and internally for 30 s using lukewarm water and non-scented, non-antibacterial Soft Soap (Colgate-Palmolive, Piscataway, NJ SKU 7418226800 LOT PS001551)) and then rinsed with water. A 30 s cleaning protocol was utilized as it is a timeframe that will most likely elicit the highest compliance rate among menstrual cup users; cleaning intervals longer than 30 s are unlikely to be followed. The test cup and negative control cup as well as the positive control cup, which was soiled but not cleaned, were each placed in a beaker with 200 ml sterile PBS and sonicated for 5 min to remove any adhered organisms (60). After sonication, aliquots were taken from the PBS and plated onto modified letheen agar with Tween (MLAT) agar. Aliquots were filtered through 0.45 µm Sterile gridded Filter Pall Microfunnel 0.45 µm Cat# 4852 as necessary to enable countable range of organisms. The *S. aureus* and *E.coli* plates were incubated for a minimum of 24 h at 35 ± 2°C (Figure 3).

### 3.5. Statistics

Analyses were performed in SAS® 9.4 software, SAS Institute, Inc., Cary, NC, USA. Each data point recovered from the sonicated cups, 200 ml PBS, were normalized against the broth and media control volume of 250 ml VDM before used for statistical analysis. Cell densities for the three cup types were compared to see if there were significant differences in organism recovery between the cups following 12 h of incubation. An ANOVA (analysis of variance) model with cup type as a fixed effect was used to analyze the log count data. Based on these analyses, data from the three cup types were combined and presented as *n* = 11 in subsequent analyses. Given the non-normality of bacteria counts, comparisons between the products were performed using log transformed data. An ANOVA model was used to compare the sonicated cup values to the media control value at the 12-h timepoint. For all comparisons, geometric mean ratios and their 95% confidence intervals are presented. All comparisons are 2-sided.

## 4. Results

### 4.1. Research question #1: determination of appropriate *in vitro* environmental conditions

#### 4.1.1. Understanding potential to introduce oxygen with Tampax menstrual cup insertion

The results of the void volume measurements and available oxygen (mg and mM) in the void volume appear in Table 1. Trace amounts of oxygen were calculated. The C-fold method had the least amount of available oxygen (0.69 mg; 0.0216 mM) compared to the punchdown fold method (1.25 mg; 0.0390 mM). Using the partial pressure from Wagner et al. (50) and the assumptions described in the methods section, time to reach low or zero O_2_ was estimated to be approximately 33 min and 1 h for the C-fold and Punchdown fold, respectively.

**Table 1:** Estimate of amount of oxygen in void volume of Tampax menstrual cup based on ideal gas law calculations and calculation of time to reach zero.

Void volume measurements		Oxygen in void volume		Estimate of remaining O_2_ by hour				Time to reach low or zero O_2_ (H:MM:SS)
Folding method	Cup volume after fold (cm^3^)	mg	mM	0	0.5	0.562	1.012	
C-fold	2.63 ± 0.50	0.69	0.0216	0.48	0.05	0.00	NA	0:33:42
Punch-down fold	4.73 ± 1.05	1.25	0.0390	0.87	0.44	0.39	0.00	1:00:42

A heavy flow Tampax menstrual cup was held upright and filled to the rim with Millipore water. The cup was subsequently folded either using the C or punchdown fold technique causing some of the water to be expelled leaving water only in the void volume (volume remaining after folding). Water remaining in the cup was measured on a 5-place analytical balance to determine void volume (mass/density = volume). The ideal gas law was used to calculate the amount of oxygen that would be present in the cup after folding using the following calculation: Ideal gas law → PV = nRT, where: *P* = 1 atmosphere, V = void volume in cup (in liters), *n* = moles of air. R = gas constant (0.08206 L·atm/K·mol), T = 310.15 K (body temperature in Kelvin). Subsequently, the time to reach low or zero O_2_ was calculated using the starting (131.00 torr) and ending (98.00 torr) partial pressure from Wagner et al. and the following assumptions: pressure remains constant [total pressure at elevated body temperature calculated using Gay Lussac's Law P1/T1 and P2/T2 where P1 is pressure of air at room temperature (1 atmosphere), T1 = 273.15 K, P2 is unknown and T2 = 310.15 K (body temperature); solving for the unknown (P2) yields 1.13 atm or 862.95 torr at 37°C], the decrease in partial pressure of O_2_ increases partial pressure of all other gasses in air, an increase in partial pressure of N_2_ and other gasses in air is proportional to volume decreases *via* P_1_V_1_ = P_2_V_2_ and the volume decrease is due to absorption of O_2_ only.

#### 4.1.2. Clinical study to determine anatomical location of menstrual cup after insertion

**Figure 4:**
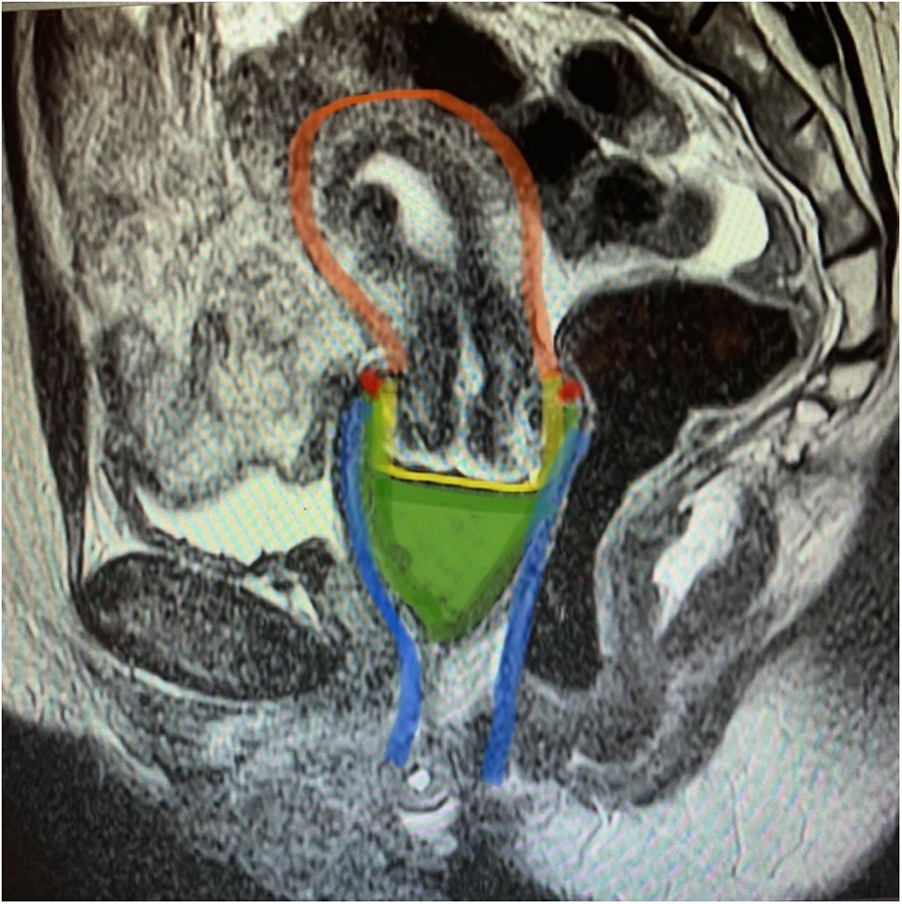
Magnetic resonance imagining of the Tampax menstrual cup in situ, sagittal view. MRI image T2 (transverse relaxation time) sagittal image of the menstrual cup in the female reproductive tract. Menstrual cup in situ contacts cervix and vaginal walls. Green: menstrual cup, blue: vagina, yellow: cervix, red: vaginal fornices, orange: uterus.

Four (4) premenopausal Caucasian women ages 34 through 43 were enrolled in the study after meeting all inclusions criteria and none of the exclusion criteria. Mean BMI score for the group was 29.7 ± 5.3 (range: 24.4–35). Images were captured with the Tampax cup (regular, *n* = 4 and heavy, *n* = 3) *in situ*. Results of the image interpretation (Figure 4) indicate that all MCs that were inserted by participants were placed in the upper part of the vagina encircling the fornices and centered under the cervix. No gaps appeared and the insertion area near the introitus was not held open for any potential air leaks with concomitant ambient oxygen entering the vagina.

The results from both the void volume measurement and the clinical study demonstrated that only a minute concentration of oxygen (C-fold: 0.69 mg and punchdown fold: 1.25 mg) would be introduced in a folded cup. This amount would dissipate quickly via vaginal tissue and would be unlikely to meaningfully change the environmental conditions of the vagina from anaerobic to aerobic. These data provided confidence that the *in vitro* study should be conducted under anaerobic conditions.

### 4.2. Research question #2: does the presence of *L. gasseri* alter the likelihood of *S. aureus* biofilm formation quantitatively or qualitatively?

#### 4.2.1. Quantitative evaluation of biofilm formation

The 3 types of medical grade silicone MCs used in this study were combined for each organism as no statistical differences (*p* < 0.05) were noted between them in terms of *L. gasseri* and *S. aureus* cell density (Table 2).

**Table 2:** Sonicated Tampax cup cell density comparisons following 12 h incubation.

			*P*-values	
Bacteria cup	Number of cups tested	Log_10_ mean (StdErr)	Comparison to prototype 1	Comparison to prototype 2
*Staphylococcus aureus*				
Tampax cup	3	2.03 (0.14)	0.8053	0.4744
Prototype 1	4	2.08 (0.12)		0.6069
Prototype 2	4	2.17 (0.12)		
Cups combined	11	2.10 (0.07)		
*Lactobacillus gasseri*				
Tampax cup	4	2.94 (0.17)	0.5426	0.9705
Prototype 1	5	3.09 (0.15)		0.4945
Prototype 2	5	2.93 (0.15)		
Cups combined	14	2.99 (0.09)		

Cell density values obtained following 12 h of incubation and sonication of the three different cups made of medical grade silicone were compared to understand if there were significant differences in organism recovery between the cups at 12 h. Original count data was log transformed prior to testing. Each data point recovered from the sonicated cups, 200 ml PBS, were normalized against the broth volume of 250 ml VDM before used for statistical analysis. An ANOVA (analysis of variance) model with cup type as a fixed effect was used to analyze the log count data.

Table 3 summarizes the results from the biofilm formation study following a 12-h incubation in a dual species culture containing *S. aureus* and *L. gasseri.* Data from media control showed *S. aureus* had grown to 10^6 ^cfu/ml and *L. gasseri* remained stable at 10^8 ^cfu/ml organisms. This was expected given the difference in cell densities of the inoculum as well as differences in the doubling time—20 min for *S. aureus* (66) and ∼150 min for *L. gasseri* (67),—between the two organisms as bacteria move from the lag phase to the low growth phase. Importantly, the *S. aureus* cell densities never grew beyond the values for *L. gasseri* during the 12 h of incubation. The values for the broth containing the MC averaged 1.0 × 10^7 ^cfu/ml *S. aureus* and were significantly higher (<0.0001) than the starting inoculum. Values obtained from the sonicated cups (1.4 × 10^2 ^cfu/ml;) after a 12 h incubation period were significantly lower (*p* < 0.0001) compared to the starting inoculum of *S. aureus* (3.2 × 10^4 ^cfu/ml), the 12 h media control [6.9 × 10^6 ^cfu/ml; (*p* < 0.001)] and the 12 h Broth [1.0 × 10^7 ^cfu/ml; (*p* < 0.0001)].

**Table 3:** Broth cell density comparison (mean cfu/ml) from Media control, broth, and sonicated Tampax cup after 12 h stagnant incubation in a dual Species culture under anaerobic conditions.

					*P*-values^a,c^		
Organism/sample	# of cups tested	Cell density (cfu/ml) mean (SE)^b^	Log_10_ cell density	Log_10_ cell density 95% confidence interval	Compare to inoculum	Compare to 12 h media control	Compare to 12 h broth
*Staphylococcus aureus*							
Inoculum (T = 0 h)	5	3.2 × 10^4^ (2.7 × 10^3^)	4.50	(4.28, 4.73)			
Media control (T = 12 h)	5	6.9 × 10^6^ (1.1 × 10^6^)	6.81	(6.58, 7.04)	<.0001*		
Broth (T = 12 h)	16	1.0 × 10^7^ (1.9 × 10^6^)	6.91	(6.78, 7.03)	<.0001*	0.4465	
Sonicated cup (T = 12 h)	11	1.4 × 10^2^ (1.8 × 10^1^)	2.10	(1.94, 2.25)	<.0001*	<.0001*	<.0001*
*Lactobaccilus gasseri*							
Inoculum (T = 0 h)	5	5.9 × 10^8^ (1.6 × 10^8^)	8.71	(8.44, 8.97)			
Media control (T = 12 h)	5	2.8 × 10^8^ (6.9 × 10^7^)	8.39	(8.12, 8.65)	0.0906		
Broth (T = 12 h)	16	3.2 × 10^8^ (5.7 × 10^7^)	8.42	(8.27, 8.57)	0.0596	0.8414	
Sonicated cup (T = 12 h)	14	1.3 × 10^3^ (2.8 × 10^2^)	2.99	(2.83, 3.15)	<.0001*	<.0001*	<.0001*

Media Control and Menstrual Cups were anaerobically incubated over 12 h in VDM-PS containing *S.aureus* + *L.gasseri*. At 12 h, samples were collected for plating and enumeration from the Media Control and Broth of each Test Product. Additionally, menstrual cups were gently rinsed with PBS before transferred to a beaker with PBS for sonication to release any adhered organisms from the menstrual cup for suspension in PBS. Organisms were plated on selective media under separate and appropriate growth conditions. The number of planktonic organisms recovered from the Broth containing menstrual cups was compared to the number of planktonic organisms recovered from the Media control. Likewise, the number of organisms recovered from the broth was compared to the organisms recovered from the sonicated cup. Each data point recovered from the sonicated cups, 200 ml PBS, were normalized against the broth volume of 250 ml VDM before used for statistical analysis. An ANOVA model with cup type as a fixed effect was used to analyze the log count data. Each experiment contained a broth control for a total *n* = 16. In some experiments the data obtained from the sonicated cup was out of countable range and in at least 1 experiment, the cup was sent for qualitative assessment via uCT and SEM. Hence, the total *n* for broth and sonicated cup will not be identical.

^a^ Cell density data were log transformed prior to statistical analysis.

^b^ Mean(cfu/ml) uses original scale.

^c^ Broth was compared to Media Control. Sonicated cup was compared to Broth.

* Denotes statistical significance at *p* < 0.05.

*L. gasseri* recovered from the menstrual cups were consistently 0.5 to 1 log higher than *S. aureus.* Results from the sonicated MCs were significantly lower when compared to the starting inoculum values (*p* < 0.0001), 12 h media control (*p* < 0.0001) and 12 h Broth (*p* < 0.0001). Although there were no significant differences for either organism when the media control was compared to the Broth, statistically significant differences were noted for both organisms that were recovered from the sonicated cups indicating very few organisms (1.4 × 10^2 ^cfu/ml *S. aureus* and 1.3 × 10^3 ^cfu/ml *L.* gasseri) adhered to the MC during the time frame of 12 h.

Results presented in Table 4 demonstrate *S. aureus* growth as a monoculture in the presence of the MC. There was an approximate 2 log increase in *S. aureus* growth on sonicated MCs when grown as a monoculture vs. when the MCs were grown in the presence of both *L. gasseri* and *S. aureus*. These differences show increased *S. aureus* bacterial attachment to menstrual cup with the same incubation time (12 h) and were significantly different from the results obtained when the test system included *L. gasseri* 2.10 log_10_ vs. 3.96 log_10;_ *p* < 0.0001 (Table 4). When the differences between the two inocula (monoculture vs. dual species culture) were examined for the media control, broth, and sonicated cup, all the differences were statistically significant (*p* < 0.0001) with increased growth in the *S. aureus* monoculture system (Table 4) demonstrating the important role of *L. gasseri* in *S. aureus* growth.

**Table 4:** Comparison of *S. aureus* cell density following 12 h incubation in a mono-culture vs. dual species culture.

Broth/starting mixture^a^	# of cups tested	Cell density (cfu/ml) mean (SE)^c^	Log_10_ cell density	Log_10_ cell density 95% confidence interval	*P*-value^b^
Inoculum (T = 0 h)					
Dual Species culture	5	3.2 × 10^4^ (2.7 × 10^3^)	4.50	(4.29, 4.72)	0.3683
Monoculture	8	2.9 × 10^4^ (6.0 × 10^3^)	4.39	(4.22, 4.56)	
Media control (T = 12 h)					
Dual Species culture	5	6.9 × 10^6^ (1.1 × 10^6^)	6.81	(6.65, 6.97)	<.0001
Monoculture	3	1.1 × 10^8^ (2.7 × 10^6^)	8.02	(7.82, 8.23)	
Broth (T = 12 h)					
Dual Species culture	16	1.0 × 10^7^ (1.9 × 10^6^)	6.91	(6.76, 7.05)	<.0001
Monoculture	5	1.4 × 10^8^ (7.8 × 10^6^)	8.14	(7.88, 8.39)	
Sonicated cup (T = 12 h)					
Dual species culture	11	1.4 × 10^2^ (1.8 × 10^1^)	2.10	(1.95, 2.25)	<.0001
Monoculture	4	1.0 × 10^4^ (2.9 × 10^3^)	3.95	(3.70, 4.20)	

^a^ Dual Species culture contained both *S.aureus* + *L.gasseri;* monoculture contained *S.aureus* alone.

^b^ Count data were log transformed prior to testing. An ANOVA model with fixed inoculum effect was used for analysis.

^c^ Mean(cfu/ml) uses original scale.

#### 4.2.2. Qualitative evaluation of biofilm formation

**Figure 5:**
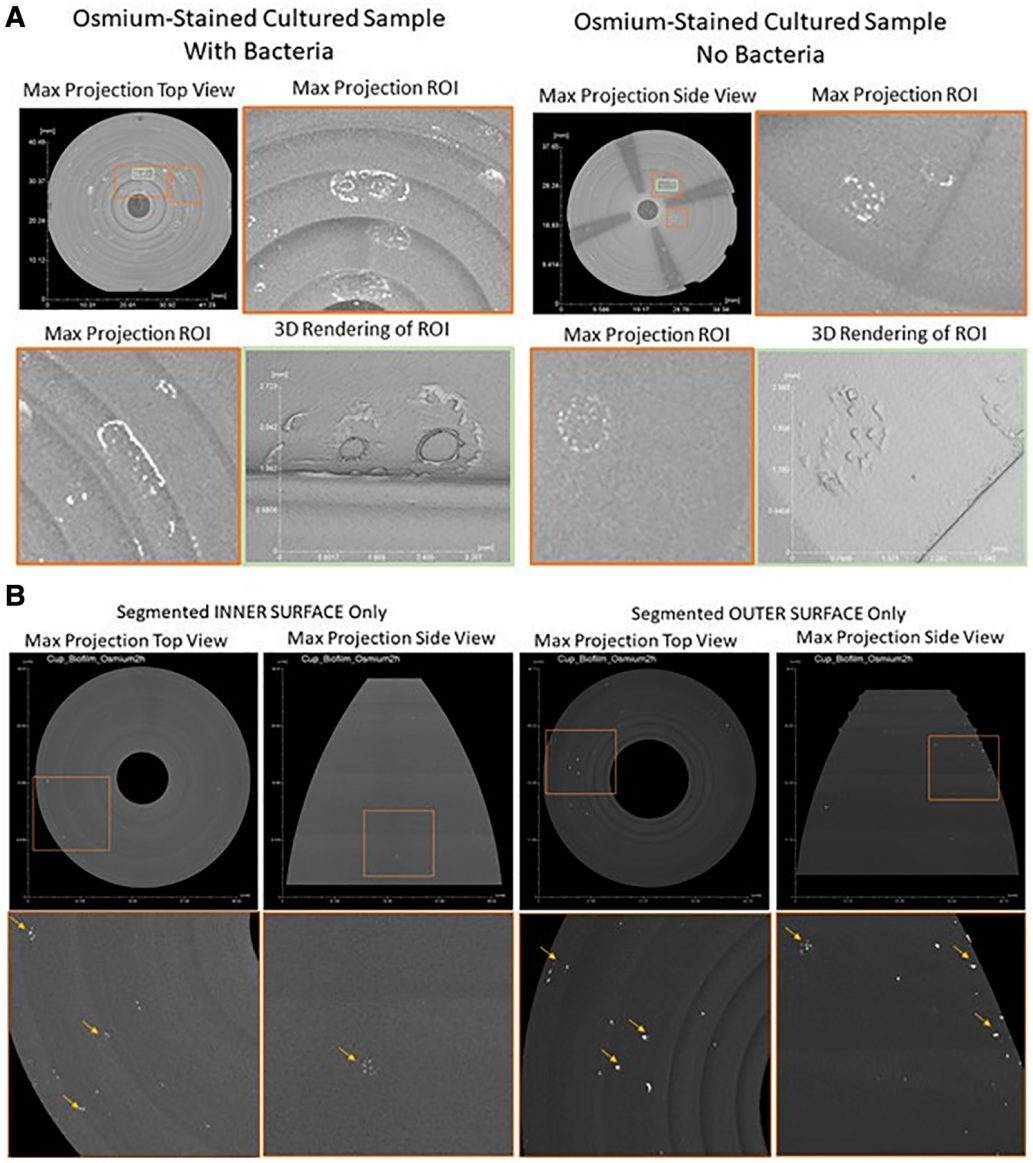
Contrast enhanced 3D x-ray imaging of Tampax menstrual cup using micro-CT. (**A**): Post-processed Tampax menstrual cup x-ray scan. By implementing morphological operations on the 3D object representing the menstrual cup, inner and outer surfaces were segmented. After segmentation, maximum projection, of both inner and outer surfaces, were acquired from top and side views [top row areas: approximate (40 mm, 37 mm)]. Bottom row represents zoomed-in regions of interest [orange squares of top row approximate areas: (11.8 mm, 12.5 mm)] showing high intensity areas (bright areas indicated by yellow arrows in bottom row) of high osmium accumulation, which areas are proxy locations for SEM imaging. (**B**): Post-processed menstrual cup x-ray scan. A maximum projection of the whole 3D object representing the menstrual cup. Regions of interest (ROIs, orange and green squares) were identified where high osmium accumulation was present as bright local areas (With Bacteria Sample, ROIs Orange Left [7.6 mm, 12.9 mm], Orange Right [10 mm, 7.7 mm], Green Center [3.6 mm, 3.95 mm]; Without Bacteria Sample, ROIs Orange Top [5.9 mm, 7.1 mm], Orange Bottom[4.7 mm, 4.8 mm], Green Center [2.35 mm, 4.8 mm]). 3D renderings of ROIs were created to depicted 3D structure and topology of high osmium accumulation per sample, with and without bacteria.

**Figure 6:**
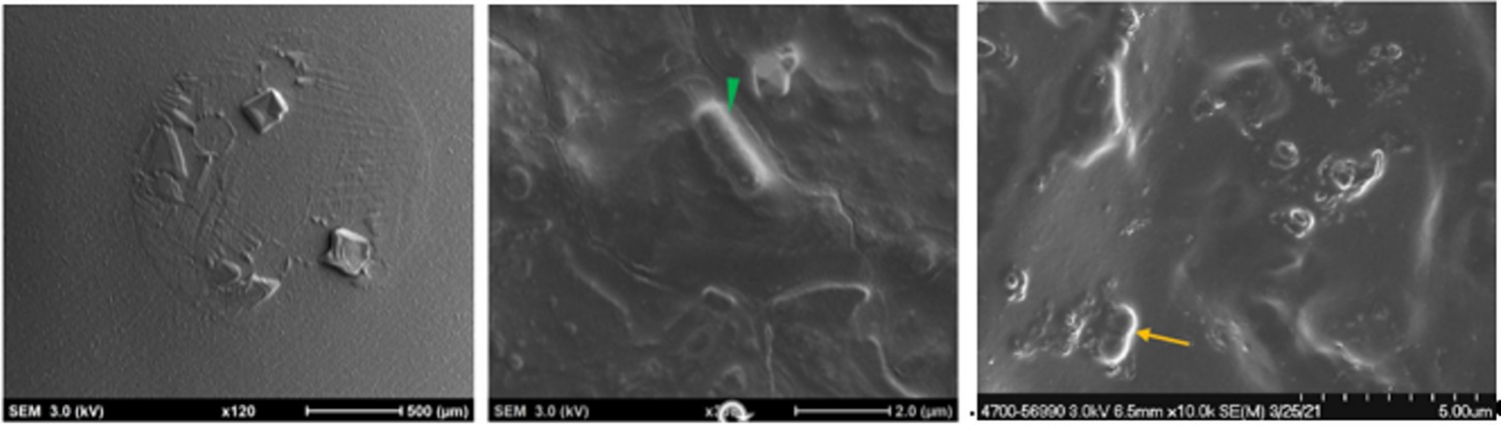
Cryogenic scanning electron microscopy images of Tampax menstrual cup following 12 h incubation with *S. aureus* and *L. gasseri*. (L-R): Low magnification cryo-SEM images of excised silicone menstrual cups (left image); higher magnification cryo-SEM images of lactobacillus (middle image, green arrowhead) and *S. aureus* (right image, yellow arrowhead).

**Figure 7:**
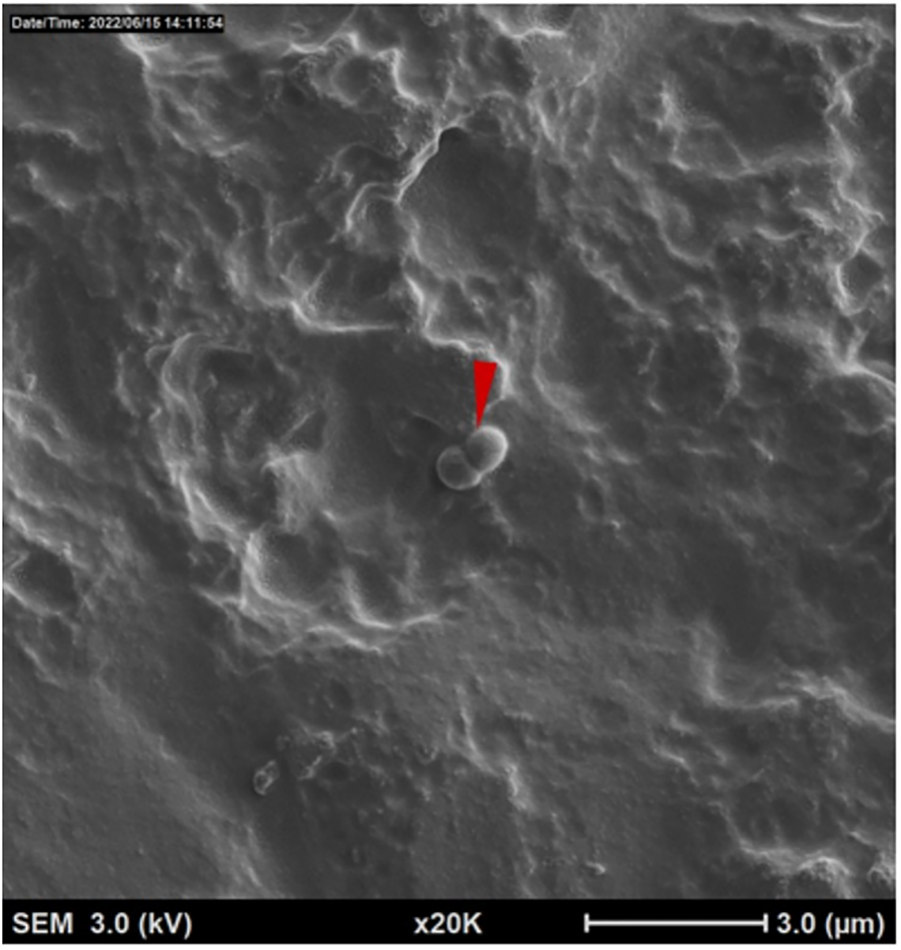
Cryogenic scanning electron microscopy images of tampax menstrual Cup following 12 h incubation with *S. aureus* alone. Low magnification cryo-SEM images of excised silicone menstrual cups of *S. aureus* (red arrowhead).

Bright areas of high osmium localization in µ-CT images served as a guide to the regions of interest (ROI) where adherent/biofilm bacteria might be found on the MC (Figures 5A,B). Bright signals were detected on the internal and external part/faces of the cup, with bacteria being identified on these surfaces following dual species incubation. Very few organisms were identified on the cup and thick biofilms, as reported in the Nonfoux, et al., could not be found (Figures 5A,B). Analysis of cryo-SEM images differentiated the morphological differences between rods (*L. gasseri)* and *S. aureus* (cocci) (Figure 6). Cryo-SEM images confirmed µ-CT observations showing very few adherent organisms were identified on the cup. Importantly, thick biofilms were not identified—only isolated adherent bacteria scattered on the surface were observed. Figure 7 provides visual evidence that *S. aureus* monoculture with the MC produced similar results, in that no biofilm was present—only a few adherent cocci organisms consistent with the morphology of *S. aureus* were identified.

### 4.3. Research question #3: Are the current cleaning instructions for the Tampax cup sufficient in removal of adherent bacteria?

**Figure 8:**
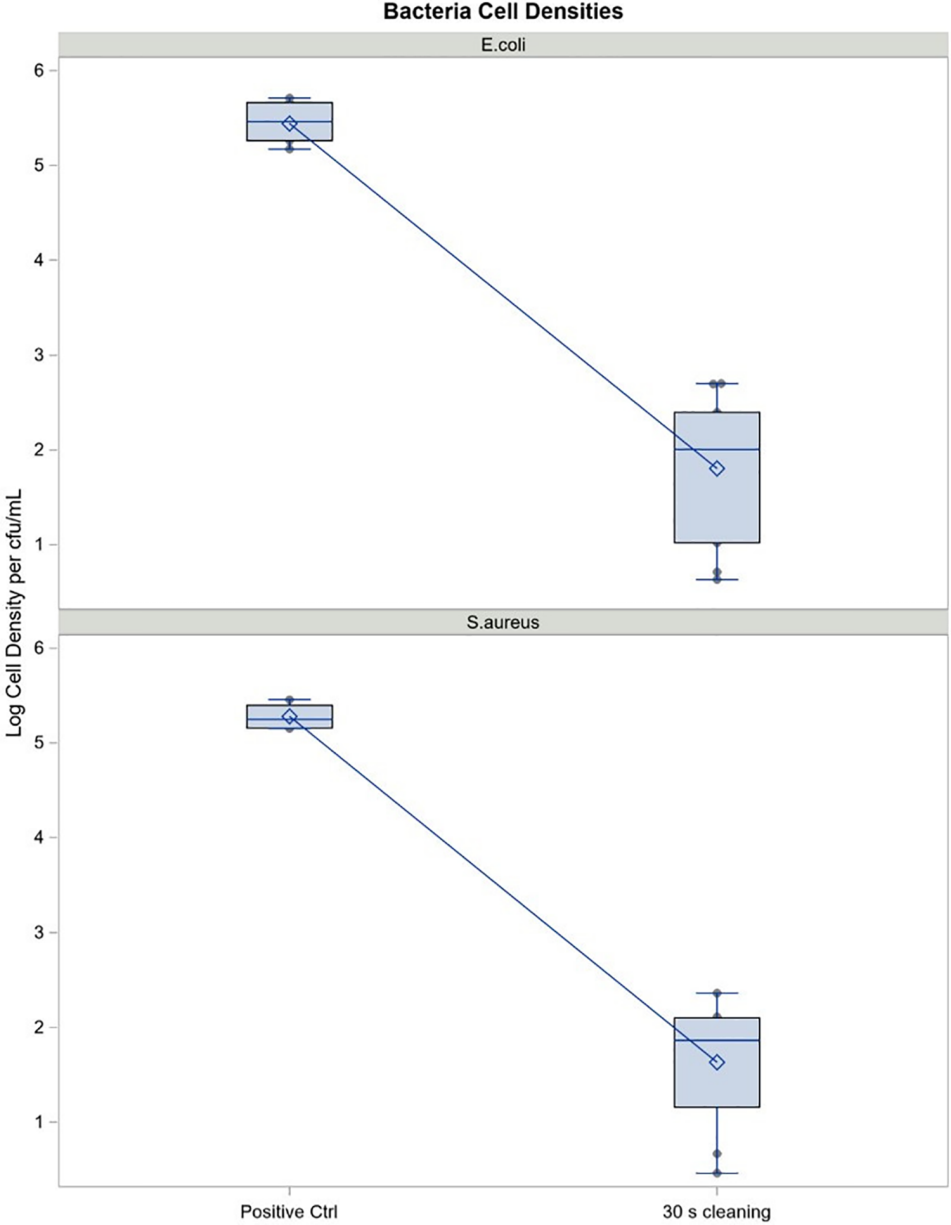
Cell density reduction of *S. aureus* and *E. coli* on the Tampax cup after 30 seconds cleaning (cfu/ml). Mean log reduction between positive control (no cleaning) and test cups (30 seconds cleaning with non-scented, non-antibacterial soap. Mean log reductions are above 3 logs, indicating a 99.9% reduction in bacterial count for both *E.coli* and *S.aureus*. *p*-values are well below 0.05, indicating that the reduction in bacterial count is significantly different from zero (no reduction).

Cell counts collected from negative control cups showed no organisms demonstrating that neither the water nor soap contributed to overall bacterial load on the MC (data not shown). Regression analysis of cfu/ml (log_10_ scale) estimated log reduction resulting from cleaning MC that had been pre-coated with nAMF, inoculated with 500 ul of 5 × 10^7 ^cfu/ml each of either *E. coli* or *S. aureus* and subsequently cleaned in lukewarm water manually with non-antibacterial, non-scented liquid soap and water for 30 s followed by a brief rinse. Log reductions in both *E. coli and S. aureus* were >3.5 logs, indicating a >99.9% reduction in bacteria compared to the initial inoculum values: reduction *p*-values < 0.0001 (Table 5). Mean log reductions between positive control (no cleaning) and test cups that were cleaned for 30 s for both organisms showed above 3 log reduction in bacterial counts (*p* < 0.05) (Figure 8). The reduction was significantly different from zero (no reduction) when compared to the uncleaned cup (*p* < 0.05).

**Table 5:** Tampax cup cell density reduction of *Staphylococcus aureus* (ATCC33589) and *Escherichia coli* (ATCC8739) after 30 seconds cleaning (cfu/ml**).**

Bacteria	Log cell count positive control	Log cell count test cup	Mean difference log reduction	SE log reduction	Reduction *p*-value	95% CI
*E. coli*	5.44 (0.24)	1.81 (0.21)	−3.64	0.32	<0.0,001*	(−4.32, −2.95)
*S. aureus*	5.28 (0.29)	1.63 (0.21)	−3.64	0.36	<0.0,001*	(−4.44, −2.84)

SE, standard error; CI, confidence interval.

Approximately 500 ul of nAMF (new artificial menstrual fluid) inoculated with 5 × 10^7^cfu/ml was applied to the menstrual cup and allowed to air dry at ambient temperature for approximately 1 h prior to testing Positive control cups (*n* = 4 and *n* = 5 for *S. aureus* and *E. coli*, respectively) were not cleaned; test cups (*n* = 8 and *n* = 9 *S. aureus* and *E. coli*, respectively) were cleaned for 30 s with a non-scented, non-antibacterial soap. An ANOVA model with cup treatment as a fixed effect was used to estimate the log reduction resulting from cleaning the menstrual cup with non-antibacterial soap and water.

* *P* < 0.05.

## 5. Discussion

Silicone materials have been available for use since the 1960s and have played an important and evolving part in products designed for the medical field. Since that time, silicone materials have been developed in various medical grades for skin and mucosal contact, as well as short-term and long-term implantation. Medical-grade silicone molding lends itself to dental and surgical applications, consumer health care products and other medical products and devices. There exists a long history of use of medical grade silicone for fabrication of external and internal devices for the human body (68–70). The first-known use of elastomeric silicone, chosen because of its stability and elasticity, was to repair a patient's bile duct in 1946 (68). The material can be cast into virtually any shape (69, 70) and its water-repellant characteristics prevent the material from sticking to wounds. Menstrual cups first became clinically available in the 1950s (71, 72). A recent review on MCs summarized available published research and concluded that they are a safe option for menstruation management and are being used internationally (37). Recent publications, by Nonfoux et al. (39) and Schlievert (73) have investigated MCs that are composed of this material in *in vitro* test systems to determine the impact of the device on *S. aureus* growth and TSST-1 production and/or biofilm formation. Results from Nonfoux et al. showed formation of *S. aureus* biofilm on the cup as well as higher levels of *S. aureus* growth and TSST-1 production compared to other intravaginal devices. The authors speculate that the menstrual cup introduced a higher volume of air into the test system providing more favorable conditions for *S. aureus.* Conversely, results from Schlievert's laboratory showed no enhanced *S. aureus* growth or TSST-1 production compared to no-device controls (73).

No single system of testing is sufficient to answer all possible questions related to risk of toxic shock syndrome, particularly as there is uncertainty about which variables are the most relevant (74). There is also no consensus on how to best test MCs and their constituent components *in vitro* to determine their potential to increase the risk of *S. aureus* growth, biofilm formation and TSST-1 production. A variety of testing systems has been described over the years (39, 73, 75) and it should be noted that the Schlievert study most closely followed the original Reiser protocol (75) (see Supplementary Table S–3). Although the Nonfoux et al., article (39) suggested it used the Reiser protocol with modifications, it seems that it only used one component, the WhirlPak bag for incubation with the menstrual cup and bacterial inoculum. It is clear that any system of laboratory testing must be reliable and reproducible and that it should have the power to detect small but significant differences between samples, even at low levels of microbial growth with the potential for subsequent biofilm formation. In vitro methods should not be susceptible to observer bias and should use culture conditions that are physiologically relevant to the human vaginal environment.

Rather than conducting two independent experiments to understand the impact of menstrual cups on *S. aureus* growth and biofilm production, the same experimental procedure was used to evaluate both endpoints. Nonfoux, et al., showed higher levels of *S. aureus* growth and toxin production with cups compared to tampons and speculated that the difference may be explained by their manipulation of the MC in the modified Reiser experimental procedure, specifically that a higher volume of air was inserted into the Whirl-Pak bag which has been shown to influence *S. aureus* growth and TSST-1 production (7, 76). Unfortunately, the authors, through placing the MC in a Whirl-Pak bag, failed to mimic the process that a woman uses to fold the MC which decreases the void space prior to vaginal insertion of the MC (Figure 1; Supplementary Table S–3). To address this, the folding and measurement of the void volume of the internal surface was modeled and determined for the amount of oxygen present. MRIs of women who inserted the MC vaginally and the images indicate that it sits in the fornices surrounding the cervix and not in the introitus where oxygen could possibly enter the vagina during use. Women with diverse BMI scores were included in the clinical study regarding placement of the MC to mimic the scores of the general population of menstruating women (77–80).

Based on the results of these experiments, an anaerobic environment was selected for testing. This approach is supported by the most recent vaginal oxygen and carbon dioxide values measured during menstruation as reported by Hill, et al., (81). Wagner (50) reported the partial pressure of oxygen within the vaginal lumen before, during and after insertion of a contraceptive diaphragm, which has similar insertion characteristics of a MC. The conclusion reached by the authors in the manuscript is that an increase in oxygen levels within the vaginal canal, due to introducing a pocket of air, creates a favorable condition for oxygen-dependent production of TSST-1 by *S. aureus.* The authors acknowledge that effects on microbial communities and especially the production of relevant toxin-like proteins was not known or evaluated during the study. In vitro TSST-1 quantification results (7) after a 12-h incubation period demonstrated significant production between 4 and 6 h and a 6-fold difference relative to anaerobic conditions. It should be noted that Wagner (50) only measured oxygen tension for 30 min after insertion which is before the first timepoint in the Schlievert study. Furthermore, electrodes (E524tc; Radiometer, Copenhagen, Denmark) that measured the oxygen were not calibrated and were susceptible to interferences. Most notably, the authors stated “…on insertion of the diaphragm, the luminal PO_2_ increased dramatically to 135 mm Hg and then decreased slowly over the next 30 min” which is inconsistent with known pressures for atmospheric gases (160 mm Hg at ambient temperature; 180 mm Hg at 37°C, body temperature). A factual error is made which states “values should be compared with a normal atmospheric PO_2_ of approximately 150 mm Hg”. The partial pressure of O_2_ at sea level is 160 mmHg (82).

Use of dual species culture of vaginal microbes for *in vitro* assays to address the potential risk of negatively impacting the human vaginal microbiome has been described in the literature (46, 47) in response to the specialized testing outlined for tampons (which are Class II medical devices, like menstrual cups) by the U.S. Food and Drug Administration (83). Herein, a novel assay was designed using quantitative and qualitative methods to evaluate the potential for MCs to develop *S. aureus* biofilms based on experimental conditions inspired by the mixed microflora assay described previously by Weissfeld (84) and Sica (38) including: boiled MCs, media designed to mimic vaginal secretions during menstruation, anaerobic incubation at relevant exposure times (up to 12-h; the manufacturer's labeled maximum time for wear) and dual species culture incubation with a vaginal lactobacillus strain in cell densities reported in the human vagina. Under these experimental conditions, designed to be simple while mimicking as much of the human physiological conditions as possible, highly reproducible results were obtained for *S. aureus* growth and did not identify the presence of *S. aureus* biofilm either quantitively or qualitatively. The methods used in these experiments meet the standards required for such an investigation to address the research questions posed at the beginning of this article.

Testing with a single organism (e.g., *S. aureus* alone) to understand growth *in vitro* does not allow for evaluating the importance of the interplay between organisms. Numerous, recent publications study growth utilizing more than one organism in a dual species methodology (85–89). Bacterial communities in the human vagina are thought to have a critical role in protecting the host against infectious disease. One of the most important mechanisms of bacteria growth inhibition is based on the presence of a lactic acid producing bacteria. In reproductive age women, it is thought they do so through the production of lactic acid resulting in a low pH environment that restricts the growth of pathogens and other opportunistic organisms (90, 91). The hallmark of vaginal health is correlated to maintaining high number of vaginal lactic acid-producing bacteria. Although there are marked differences in the species composition and rank-abundances of populations in vaginal bacterial communities (12, 92, 93) among women, it appears that almost all are dominated by homofermentative lactic acid bacteria (93). In the vagina, the keystone species, (e.g., *L. gasseri)* produces lactic acid through the fermentation of carbohydrates which results in a drop in pH and an inhibition of *S. aureus* growth. Creating a low pH in the vaginal environment through the production of organic acids may be conserved despite differences in the bacterial species present in the various communities identified in the human vagina.

Multiple publications have noted the specific factors that influence growth inhibition of *S aureus* when Lactobacilli spp, and specifically *L. gasseri*, is grown in chemically defined medium. The most prominent reason for the growth inhibition was published by Elkins, et al. (49), indicating that *Lactobacillus*-mediated *S. aureus* growth inhibition phenomenon was via acidification of the vaginal environment. It could be multifactorial considering some isolated activity in certain strains were observed in spent culture supernatant potentially indicating some bacteriocin-type production. Since the vast majority of lactobacilli required live cultures to exhibit inhibition, this characteristic implies a more general phenomenon indicative of acidity of the environment being the prime driver. Elkins et al. (49), noted that *L. gasseri* reduced the pH of the medium through fermentation of the glucose in the medium and that other factors were produced, but most specifically focus on the fermentation of the carbohydrates. (Of note, reference 49 utilized the same ATCC strain in their study as was used in in the current study; results showed the *L. gasseri* dropped the pH.) Boskey et al. (94), showed that during exponential growth, Lactobacillus species acidified CDM growth medium *in vitro* at rates on the order of 10^6^ protons/bacterium(s) supporting the idea that the acidity of the vagina is predominantly produced and regulated by this keystone species. Finally, Dufresne et al. (95), in investigating mTSS using a dual species system of *L. gasseri* and *S. aureus in vitro*, noted that increased glucose concentration in a vaginally defined medium could influence *S. aureus* physiologically when tested in an *in vitro* vagina model system using a chemically defined medium referred to as VDM. Given this, testing in a dual species system is appropriate when evaluation growth *in vitro* using VDM.

Lactic acid (protonated lactate) has broad antimicrobial activity and vaginal lactobacilli producing lactic acid are known to confer protection against reproductive tract infections when they are predominant in the vaginal microbiota (96). Also, lactobacilli have been noted to produce biosurfactants (97) and Santos, et al. (98), reviewed the presence of microbial biosurfactant which constitute a diverse group of surface-active molecules. They are amphiphilic compounds with both hydrophilic and hydrophobic moieties and with a distinct tendency to accumulate at the interfaces of materials (99). Biosurfactants from various species of lactobacilli have demonstrated anti-biofilm activity against *S. aureus* strains (100, 101) and have interfered with bacteria and yeasts adherence to silicone rubber (102). Lactic acid bacteria antagonism to *S. aureus* may act at more than one level by either hampering its growth rate and or by interfering with the modulation of virulence factors such as production of superantigens like TSST-1. Although not investigated in this study, some investigators have noted a modulating effect on staphylococcal enterotoxins/superantigen production in mixed cultures (103–105). Also noteworthy is that some bacteria produce sophorlipids that have activity against microbial biofilms on medical-grade silicone (106).

Construct validity is the accumulation of evidence to support the interpretation of what a measure reflects. Hence, it concerns the extent to which the test or measure used accurately assesses what it is supposed to evaluate. In research, it is important to operationalize constructs into concrete and measurable characteristics based on one's idea of the construct and its dimensions. The *in vitro* study designed and utilized in this report is most relevant to address the complexity of studying any potential product/device impact in a complex environment such as the human vagina during menstruation -specifically, the media selection that can mimic the characteristics of human menstrual fluid, anaerobic conditions of the human vagina, paucity of oxygen in a folded cup prior to insertion as well as selection of test strains that have a pedigree of being sourced from the human vagina and prepared in relevant cell densities found in the human vagina. The strains are available through the American Type and Culture Collection (Mannasas, VA, USA) for use by all qualified researchers who apply to obtain them.

The term biofilm describes well established consortium of cells attached to the surface. Formation of biofilms starts with individual cells attaching to the surface, and that stage is usually called initial attachment, and it may lead to biofilm formation. The quantitative and qualitative data from the *S. aureus* biofilm formation study presented in this paper demonstrated that: (1) very few organisms of the mixed culture adhered to the MC; (2) *S. aureus* was rarely identified and;(3) the predominant morphology of adherent bacteria were rod-shaped bacteria consistent with the morphology of *L. gasseri* used in the test system. Biofilm formation is a social group behavior. Each of the steps from initial attachment to the dispersion and transmission of mature biofilm is strictly controlled by multiple regulatory systems or regulators and to develop a young biofilm *in vitro* may take several days (107, 108). It should be noted that biofilm formation was not observed by either of the two test organisms on the MC during SEM visualization. These data support the importance of the presence of *L. gasseri* in controlling the growth and adherence of *S. aureus* to the MC. It is hypothesized that the results from this *in vitro* system are similar to what would occur in the human vagina when using a MC during menstruation. It is suggested that any future studies involve gentle movement of the test systems to model human movement.

MCs are a comfortable, safe (when used as directed) and efficient option for menstrual hygiene (109). They are marketed in almost 100 different countries and are viewed as a meaningful alternative for use compared to disposable menstrual hygiene products. Different MC brands recommend a variety of techniques for cleaning in-between uses and storage from month to month. Overall, the cleaning directions vary and range from wiping the cup to boiling for 10 min (110). As noted in the same 2021 report, “[m]anufacturers should standardize cleaning terminology to internationally agreed upon definitions” as the terms *sterilization*, *disinfection* and *sanitization* are used interchangeable and inconsistently in directions for caring for the MC (111). The term “cleaning” may be more appropriate for the MC as it involves removal of visible organic and inorganic material from objects as well as a significant number of bacteria from surfaces that is normally accomplished manually using water with detergents. Cleaning is a necessary step prior to subjecting devices to boiling. The methodology described herein utilizes slightly different design parameters than described by Wunsch (40) to understand cleaning effectiveness including use of an artificial menstrual fluid, meant to represent the complex nature of menses (Supplementary Table S–4). The data described herein have demonstrated that, when the cleaning protocol was initiated, the soiled MC with added bacteria using model gram positive and gram negative organisms, had at least a 3 log reduction (99.9%) when *S. aureus* and *E. coli* were inoculated on the MC compared to study initiation at t = 0 h. Less than 200 cfu/ml from the MC were identified as part of the sonication protocol. It can be concluded that few organisms adhered to the MC following the cleaning protocol using unscented non-antibacterial soap, water, and manual scrubbing with fingers (and subsequent rinsing) for at least 30 s. These data suggest cleaning the MC with soap and water is an effective technique at removing bacteria between uses during the menstrual cycle. This degree of bioburden reduction (99.9%) achieved in this study is considered the gold standard for cleaning single-consumer reusable devices (43) and has been deemed acceptable by regulatory authorities (112) in the clearance of other reusable, intravaginal devices (e.g., Fem Cap) similar to the menstrual cup.

The processes governing bacterial adherence and subsequent biofilm formation are rather complex, involving several steps under the right environmental conditions and exposure time. When all these co-factors are met, almost all medical device surfaces are susceptible to colonization by adherent bacteria during human use (99, 113). Nevertheless, the results presented from our experiments do not support Nonfoux, et al. (39), regarding *S. aureus* biofilm formation on MC or Wunch et al. (40), regarding cleaning the MC. No visual identification of any biofilm formation by *S. aureus* using cryo-SEM was observed a well-accepted methodology for visualizing microbes and their modality of growth. As summarized by Schlievert in the 2020 *in vitro* study, “…devices such as diaphragms, menstrual discs, and menstrual cups, which are not absorbent, do not increase TSST-1 production vs. no-device controls”. Given this, Schlievert suggests that the rare reports of mTSS with the MC may be more coincidental than as a risk factor for the disease when the device is used as recommended (73). It is clear, based on these studies and previous reports that the current recommendations to care and manage the Tampax MC between uses are appropriate and provide consumers with an easily accessible method for cleaning the cup between uses during the menstrual cycle. The conclusion is that this work provides data counter to data published by Nonfoux et al. (39), that called into question the safety of the Tampax® cup during use by menstruating women. Based on the experimental results provided herein, the data support the continued safe use of the Tampax® cup when used and maintained as recommended.

## Data Availability

The original contributions presented in the study are included in the article/**Supplementary Material**, further inquiries can be directed to the corresponding author/s.

## References

[1] Novick RP. Autoinduction and signal transduction in the regulation of staphylococcal virulence. Mol Microbiol. (2003) 48(6):1429–49. 10.1046/j.1365-2958.2003.03426x 12791129

[2] Parsonnet J Hansmann MA Delaney ML Modern PA Dubois AM Wieland-Alter W Prevalence of toxic shock syndrome toxin 1-producing *Staphylococcus aureus* and the presence of antibodies to this superantigen in menstruating women. J Clin Microbiol. (2005) 43(9):4628–34. 10.1128/JCM.43.9.4628-4634.2005 16145118 PMC1234102

[3] Parsonnet J Hansmann MA Seymour JL Delaney ML Dubois AM Modern PA Persistence survey of toxic shock syndrome toxin-1 producing *Staphylococcus aureus* and serum antibodies to this superantigen in five groups of menstruating women. BMC Infect Dis. (2010) 10:249. 10.1186/1471-2334-10-249 20731864 PMC2936898

[4] Kansal R Davis CC Hansmann M Seymour J Parsonnet J Modern P Structural and functional properties of antibodies to the superantigen TSST-1 and their relationship to menstrual toxic shock syndrome. J Clin Immunol. (2007) 27(3):327–38. 10.1007/s10875-007-9072-4 17340193

[5] Schlievert PM Davis CC. Device-associated menstrual toxic shock syndrome. Clin Microbiol Rev. (2020) 33(3):1–42. 10.1128/CMR.00032-19 PMC7254860 32461307

[6] Yarwood JM Schlievert PM. Oxygen and carbon dioxide regulation of toxic shock syndrome toxin 1 production by *Staphylococcus aureus* MN8. J Clin Microbiol. (2000) 38:1797–803. 10.1128/JCM.38.5.1797-1803.2000 10790102 PMC86591

[7] Schlievert PM Blomster DA. Production of staphylococcal pyrogenic exotoxin type C: influence of physical and chemical factors. J Infect Dis. (1983) 147(2):236–42. 10.1093/infdis/147.2.236 6827140

[8] Schlievert PM Blomster DA Kelly JA. Toxic shock syndrome *Staphylococcus aureus:* effect of tampons on toxic shock syndrome toxin 1 production. Obstet Gynecol. (1984) 64(5):666–71 6436761

[9] Döderlein A. Das scheidensekret und seine bedeutung fur puerperalfieber. Zentralblatt fur Bakteriology. (1892) 11:1–86.

[10] Hickey RJ Zhou X Pierson JD Ravel J Forney LJ. Understanding vaginal microbiome complexity from an ecological perspective. Transl Res. (2012) 160(4):267–82. 10.1016/j.trsl.2012.02.008 22683415 PMC3444549

[11] Dasari S. Recent findings of Lactobacillus diversity and their functional role in vaginal ecosystems in recent development in applied microbiology and biochemistry. Bloomington, Indiana: Academic Press (2019). 3–12.

[12] Ravel J Gajer P Abdo Z Schneider GM Koenig S McCulle SL Vaginal microbiome of reproductive-age women. Proc Natl Acad Sci U S A. (2011) 108(Suppl 1):4680–7. 10.1073/pnas.1002611107 20534435 PMC3063603

[13] Aldunate M Tyssen D Johnson A Zakir T Sonza S Moench T Vaginal concentrations of lactic acid potently inactivate HIV. J Antimicrob Chemother. (2013) 68(9):2015–25. 10.1093/jac/dkt156 23657804 PMC3743514

[14] Ma B Forney LJ Ravel J. Vaginal microbiome: rethinking health and disease. Annu Rev Microbiol. (2012) 66:371–89. 10.1146/annurev-micro-092611-150157 22746335 PMC3780402

[15] Greenbaum S Greenbaum G Moran-Gilad J Weintraub AY. Ecological dynamics of the vaginal microbiome in relation to health and disease. Am J Obstet Gynecol. (2019) 220(4):324–35. 10.1016/j.ajog.2018.11.1089 30447213

[16] Foster JA Krone SM Forney LJ. Application of ecological network theory to the human microbiome. Interdiscip Perspect Infect Dis. (2008) 2008:839501. 10.1155/2008/839501 19259330 PMC2648623

[17] Lamont RF Sobel JD Akins RA Hassan SS Chaiworapongsa T Kusanovic JP The vaginal microbiome: new information about genital tract flora using molecular based techniques. BJOG. (2011) 118(5):533–49. 10.1111/j.1471-0528.2010.02840.x 21251190 PMC3055920

[18] Valenti P Rosa L Capobianco D Lepanto MS Schiavi E Cutone A Role of lactobacilli and lactoferrin in the mucosal cervicovaginal defense. Front Immunol. (2018) 9:376. 10.3389/fimmu.2018.00376 29545798 PMC5837981

[19] MacPhee RA Miller WL Gloor GB McCormick JK Hammond JA Burton JP Influence of the vaginal microbiota on toxic shock syndrome toxin 1 production by *Staphylococcus aureus*. Appl Environ Microbiol. (2013) 79(6):1835–42. 10.1128/AEM.02908-12 23315732 PMC3592239

[20] Hütt P Lapp E Štšepetova J Smidt I Taelma H Borovkova N Characterisation of probiotic properties in human vaginal lactobacilli strains. Microb Ecol Health Dis. (2016) 27:30484. 10.3402/mehd.v27.30484 27527701 PMC4985617

[21] Strus M Brzychczy-Wloch M Gosiewski T Kochan P Heczko PB. The *in vitro* effect of hydrogen peroxide on vaginal microbial communities. FEMS Immunol Med Microbiol. (2006) 48(1):56–63. 10.1111/j.1574-695X.2006.00120.x 16965352

[22] Cherian PT Wu X Maddox MM Singh AP Lee RE Hurdle JG. Chemical modulation of the biological activity of reutericyclin: a membrane-active antibiotic from *Lactobacillus reuteri*. Sci Rep. (2014) 4:4721. 10.1038/srep04721 24739957 PMC4894453

[23] Schlievert PM Kilgore SH Benavides A Klingelhutz AJ. Pathogen stimulation of interleukin-8 from human vaginal epithelial cells through CD40. Microbiol Spectr. (2022) 10(2):e0010622. 10.1128/spectrum.00106-22 35297656 PMC9045207

[24] Murray EJ Crowley RC Truman A Clarke SR Cottam JA Jadhav GP Targeting *Staphylococcus aureus* quorum sensing with nonpeptidic small molecule inhibitors. J Med Chem. (2014 Mar 27) 57(6):2813–9. 10.1021/jm500215s Epub 2014 Mar 13. PMID: 24592914; PMCID: PMC4010551. 24592914 PMC4010551

[25] Bjarnsholt T Alhede M Alhede M Eickhardt-Sorensen SR Moser C Kuhl M The in vivo biofilm. Trends. Microbiol. (2013) 21(9):466–74. 10.1016/j.tim.2013.06.002 23827084

[26] Donlan RM. 15 October biofilm formation: a clinically relevant microbiological process. Clin Infect Dis. (2001) 33(8):1387–92. 10.1086/322972 11565080

[27] Costerton JW Geesey GG Cheng KJ. How bacteria stick. Sci. Am. (1978) 238(1):86–95. 10.1038/scientificamerican0178-86 635520

[28] Yarwood JM Bartels DJ Volper EM Greenberg EP. Quorum sensing in *Staphylococcus aureus* biofilms. J Bacteriol. (2004) 186(6):1838–50. 10.1128/JB.186.6.1838-1850.2004 14996815 PMC355980

[29] Singh R Ray P. Quorum sensing-mediated regulation of staphylococcal virulence and antibiotic resistance. Future Microbiol. (2014) 9(5):669–81. 10.2217/fmb.14.31 24957093

[30] Mardh PA Westrom L. Adherence of bacteria to vaginal epithelial cells. Infect Immun. (1976) 13(3):661–6. 10.1128/iai.13.3.661-666.1976 5372 PMC420661

[31] Veeh RH Shirtliff ME Petik JR Flood JA Davis CC Seymour JL Detection of *Staphylococcus aureus* biofilm on tampons and menses components. J Infect Dis. (2003) 199(4):519–30. 10.1086/377001 12898438

[32] Gould FG Carey MP Plummer EL Murray GL Danielewski JA Tabrizi SN Bacterial biofilm formation on vaginal ring pessaries used for pelvic organ prolapse. Int Urogynecol J. (2022) 33(2):287–95. 10.1007/s00192-021-04717-x 33660005

[33] Averette HE Weinstein GD Frost P. Autoradiographic analysis of cell proliferation kinetics in human genital tissues. I. Normal cervix and vagina. Am J Obstet Gynecol. (1970) 108(1):8–17. 10.1016/0002-9378(70)90195-x 5454588

[34] Anderson J Paterek E. Vaginal foreign body evaluation and treatment. In: Statpearls. Treasure Island (FL): StatPearls Publishing (2022). 31747201

[35] Chalmers LW. The menstrual cup. The intimate Side of a Woman's Life. Radio City, New York: Pioneer Publications, Inc. (1937).

[36] Hait A Powers SE. The value of reusable feminine hygiene products evaluated by comparative environmental life cycle assessment. Resour Conserv Recycl. (2019) 150:e104422. 10.1016/j.resconrec.2019.104422

[37] van Eijk AM Zulaika G Lenchner M Mason L Sivakami M Nyothach E Menstrual cup use, leakage, acceptability, safety, and availability: a systematic review and meta-analysis. Lancet Public Health. (2019) 4(8):e376–93. 10.1016/S2468-2667(19)30111-2 31324419 PMC6669309

[38] Sica VP Friberg M Teufel A Streicher-Scott J Hu P Sauer U Safety assessment scheme for menstrual cups and application for the evaluation of a menstrual cup comprised of medical grade silicone. EBioMedicine. (2022) 86:104339. 10.1016/j.ebiom.2022.104339 36370636 PMC9664401

[39] Nonfoux L Chiaruzzi M Badiou C Baude J Tristan A Thioulouse J Impact of currently marketed tampons and menstrual cups on *Staphylococcus aureus* growth and toxic shock syndrome toxin 1 production in vitro. Appl Environ Microbiol. (2018) 84(12):e00351–18. 10.1128/AEM.00351-18 29678918 PMC5981080

[40] Wunsch N Green SJ Adam S Hampton J Phillips-Howard PA Mehta SD. In vitro study to assess effective cleaning techniques for removing *Staphylococcus aureus* from menstrual cups. Int J Environ Res Public Health. (2022) 19(3):1450. 10.3390/ijerph19031450 35162481 PMC8835062

[41] United States Pharmacopeia—National Formulary. General Chapter <88> Biological reactivity tests, in vivo. (2020). Available at: http://www.uspbpep.com/usp29/v29240/usp29nf24s0_c88.html (Accessed 27 August 2022)

[42] ANSI/AAMI TIR12. Designing, testing and labeling reusable medical devices for reprocessing in health care facilities: a guide for medical device manufacturers (2010).

[43] AAMI TIR30. 2011/(R)2016. A compendium of processes, material, test methods, and acceptance criteria for cleaning reusable medical devices).

[44] ANSI/AAMI/ISO 17664. (2017). Processing of health care products—information to be provided by the medical device manufacturer for the processing of medical devices.

[45] Geshnizgani AM Onderdonk AB. Defined medium simulating genital tract secretions for growth of vaginal microflora. J Clin Microbiol. (1992) 30:1323–6. 10.1128/jcm.30.5.1323-1326.1992 1583140 PMC265277

[46] Weissfeld AS. Development of an in vitro vaginal microflora assay to evaluate safety of tampons. Obstet Gynecol. (2002) 99:40S. Papers on Current Clinical and Basic Investigations. 10.1016/S0029-7844(02)01754-4

[47] Gad SC. Special studies—tampons. Safety evaluation of medical devices. Switzerland: Marcel Deckker, Inc. Basel (2002). 499–500.

[48] Flood JA Tripp TJ Davis CC Hill DR Schlievert PM. A toroid model for in vitro investigations of toxic shock syndrome toxin-1 production. J Microbiol Methods. (2004) 57:283–8. 10.1016/j.mimet.2004.01.005 15063069

[49] Elkins CA Muñoz ME Mullis LB Stingley RL Hart ME. Lactobacillus-mediated inhibition of clinical toxic shock syndrome *Staphylococcus aureus* strains and its relation to acid and peroxide production. Anaerobe. (2008) 14(5):261–7. 10.1016/j.anaerobe.2008.08.003 18926917

[50] Wagner G Levin RJ Bohr L. Diaphragm insertion increases human vaginal oxygen tension. Am J Obstet Gynecol. (1988) 158(5):1040–3. 10.1016/0002-9378(88)90214-1 3369481

[51] Robb WL. Thin silicone membranes–their permeation properties and some applications. Ann N Y Acad Sci. (1968) 146(1):119–37. 10.1111/j.1749-6632.1968.tb20277.x 5238627

[52] Dutta A Popel AS. A theoretical analysis of intracellular oxygen diffusion. J Theor Biol. (1995) 176(4):433–45. 10.1006/jtbi.1995.0211 8551742

[53] Pias SC. Pathways of oxygen diffusion in cells and tissues: hydrophobic channeling via networked lipids. Adv Exp Med Biol. (2020) 1232:183–90. 10.1007/978-3-030-34461-0_23 31893409 PMC7302104

[54] Boveris DL Boveris A. Oxygen delivery to the tissues and mitochondrial respiration. Front Biosci. (2007) 12:1014–23. 10.2741/2121 17127356

[55] Pias SC. How does oxygen diffuse from capillaries to tissue mitochondria? Barriers and pathways. J Physiol. (2021) 599(6):1769–82. 10.1113/JP278815 33215707

[56] Thiyagarajan DK Basit H Jeanmonod R. Physiology, menstrual cycle. In: Statpearls. Treasure Island (FL): StatPearls Publishing (2023). Available at: https://www.ncbi.nlm.nih.gov/books/NBK500020/ (Updated 2022 Oct 24). 29763196

[57] Code of Federal Regulations TITLE 45 PUBLIC WELFARE Department of Health and Human Services PART 46 PROTECTION OF HUMAN SUBJECTS Revised January 15, 2009, Effective July 14, 2009. 11686173

[58] Johnson SR Petzold CR Galask RP. Qualitative and quantitative changes of the vaginal microbial flora during the menstrual cycle. Am J of Repro Immun and Micro. (1985) 9(1):1–5. 10.1111/j.1600-0897.1985.tb00331.x 4051082

[59] Redondo-Lopez V Cook RL Sobel JD. Emerging role of lactobacilli in the control and maintenance of the vaginal bacterial microflora. Rev Infec Dis. (1990) 12(5):856–72. 10.1093/clinids/12.5.856 2237129

[60] Haaber J Cohn MT Petersen A Ingmer H. Simple method for correct enumeration of *Staphylococcus aureus*. J Microbiol Methods. (2016) 125:58–63. 10.1016/j.mimet.2016.04.004 27080188

[61] Niehaus WL Howlin RP Johnston DA Bull DJ Jones GL Calton E Development of x-ray micro-focus computed tomography to image and quantify biofilms in central venous catheter models in vitro. Microbiology (Reading). (2016) 162(9):1629–40. 10.1099/mic.0.000334 27384949

[62] Sawyer LC Grubb DT Meyers GF. Ch 4: Specimen preparation methods. Polymer microscopy. 3rd edn. New York: Springer (2008). https://link.springer.com/content/pdf/10.1007/978-0-387-72628-1.pdf?pdf=button

[63] Wildenschild D Sheppard AP. X-ray imaging and analysis techniques for quantifying pore-scale structure and processes in subsurface porous medium systems. Adv Water Resour. (2013) 51:217–46. ISSN 0309-1708. 10.1016/j.advwatres.2012.07.018

[64] Wilson C Lukowicz R Merchant S Valquier-Flynn H Caballero J Sandoval J Quantitative and qualitative assessment methods for biofilm growth: a Mini-review. Res Rev J Eng Technol. (2017) 6(4):1–42. http://www.rroij.com/open-access/quantitative-and-qualitative-assessment-methods-for-biofilm-growth-a-minireview-.pdf PMC6133255 30214915

[65] Azeredo J Azevedo NF Briandet R Cerca N Coenye T Costa AR Critical review on biofilm methods. Crit Rev Microbiol. (2017) 43(3):313–51. Received 17 Mar 2016, Accepted 28 Jun 2016, Published online: 21 Nov 2016 https://www.tandfonline.com/doi/full/10.1080/1040841X.2016.1208146# 10.1080/1040841X.2016.1208146 27868469

[66] Missiakas DM Schneewind O. Growth and laboratory maintenance of *Staphylococcus aureus*. Curr Protoc Microbiol. (2013):1–12. 10.1002/9780471729259.mc09c01s28 Chapter 9:Unit 9C.1. PMID: 23408134; PMCID: PMC6211185. PMC6211185 23408134

[67] Trojok R. (2013). Towards the utilization of lactic acid bacteria as contraceptive agents Project title: “The Liquid Condom Project”. Available at: https://www.researchgate.net/figure/Doubling-time-with-standard-deviation-of-Lcrispatus-L-gasseri-L-jensenii-L_fig4_258205987 (Accessed 19 Mar, 2023)

[68] Apalakis A. An experimental evaluation of the types of material used for bile duct drainage tubes. Br J Surg. (1976) 63(6):440–5. 10.1002/bjs.1800630608 1276671

[69] Colpani A Fiorentino A Ceretti E. Design and fabrication of customized tracheal stents by additive manufacturing. Procedia Manuf. (2020) 47:1029–35. 10.1016/j.promfg.2020.04.318

[70] Colas A Curtis J. Biomaterial science, medical application of silicones in an Introduction to materials in medicine. 2nd edn. Elsevier Academic Press). 698. Chapter 7.19. (2005) p. 80–6.

[71] Liswood R. Internal menstrual protection; use of a safe and sanitary menstrual cup. Obstet Gynecol. (1959) 13:539–43. 13644862

[72] Pena EF. Menstrual protection. Advantages of the menstrual cup. Obstet Gynecol. (1962) 19:684–7. 14485045

[73] Schlievert PM. Effect of non-absorbent intravaginal menstrual/contraceptive products on *Staphylococcus aureus* and production of the superantigen TSST-1. Eur J Clin Microbiol Infect Dis. (2020) 39(1):31–8. 10.1007/s10096-019-03685-x 31853743

[74] Parsonnet J Modern PA Giacobbe KD. Effect of tampon composition on production of toxic shock syndrome toxin-1 by *Staphylococcus aureus* in vitro. J Infect Dis. (1996) 173(1):98–103. 10.1093/infdis/173.1.98 8537689

[75] Reiser RF Hinzman SJ Bergdoll MS. Production of toxic shock syndrome toxin 1 by *Staphylococcus aureus r*estricted to endogenous air in tampons. J Clin Microbiol. (1987) 25(8):1450–2. 10.1128/jcm.25.8.1450-1452.1987 3624443 PMC269245

[76] Recsei P Kreiswirth B O'Reilly M Schlievert P Gruss A Novick R. Regulation of exoprotein gene expression in *Staphylococcus aureus* by *agr*. Mol Gen Genet. (1986) 202(1):58–61. 10.1007/BF00330517 3007938

[77] Abramowitz MK Hall CB Amodu A Sharma D Androga L Hawkins M. Muscle mass, BMI, and mortality among adults in the United States: a population-based cohort study. PLoS One. (2018) 13(4):e0194697. 10.1371/journal.pone.0194697; Erratum in: PLoS One. 2018 May 24;13(5):e0198318. 29641540 PMC5894968

[78] Banack HR Wactawski-Wende J Hovey KM Stokes A. Is BMI a valid measure of obesity in postmenopausal women? Menopause. (2018) 25(3):307–13. 10.1097/GME.0000000000000989 29135897 PMC5821529

[79] Blew RM Sardinha LB Milliken LA Teixeira PJ Going SB Ferreira DL Assessing the validity of body mass index standards in early postmenopausal women. Obes Res. (2002) 10(8):799–808. 10.1038/oby.2002.108 12181389

[80] MacDonald CJ Madika AL Lajous M Laouali N Artaud F Bonnet F. Associations between physical activity and incident hypertension across Strata of body mass Index: a prospective investigation in a large cohort of French women. J Am Heart Assoc. (2020) 9(23):e015121. 10.1161/JAHA.119.015121 33190573 PMC7763781

[81] Hill DR Brunner ME Schmitz DC Davis CC Flood JA Schlievert PM In vivo assessment of human vaginal oxygen and carbon dioxide levels during and post menses. J Appl Physiol (1985). (2005) 99(4):1582–91. 10.1152/japplphysiol.01422.2004 15932958

[82] Haynes WM Lide DR Bruno TJ. CRC Handbook of chemistry and physics: a ready-reference book of chemical and physical data. 2016–2017. 97th edn. Boca Raton, FL: CRC Press (2016).

[83] CDRH. Draft Guidance for the Content of Premarket Notification for Menstrual Tampons, Obstetrics-Gynecology Devices Branch, ODE, CDRH, U.S. Food and Drug Administration May 25. Menstrual Tampons and Pads: Information for Premarket Notification Submissions (510(k)s)—Guidance for Industry and FDA Staff | FDA. (Accessed February 8, 2023) (1995).

[84] Weissfeld A. The history of tampons: from ancient times to an FDA regulated device. Clin Micro Newsletter. (2010) 32(10):2010. 10.1016/j.clinmicnews.2010.04.003

[85] Kumawat KC Sharma P Nagpal S Gupta RK Sirari A Nair RM Dual microbial inoculation, a game changer?—bacterial biostimulants with multifunctional growth promoting traits to mitigate salinity stress in spring mungbean. Front Microbiol. (2021) 11:600576. 10.3389/fmicb.2020.600576 33584566 PMC7874087

[86] Montelongo-Jauregui D Srinivasan A Ramasubramanian AK Lopez-Ribot JL. An in vitro model for oral mixed biofilms of *Candida albicans* and *Streptococcus gordonii* in synthetic saliva. Front Microbiol. (2016) 7:686. 10.3389/fmicb.2016.00686 27242712 PMC4864667

[87] Schlüter A Sczyrba A Maestri E Marmiroli N Neuhoff D Nesme J Identification of beneficial microbial consortia and bioactive compounds with potential as plant biostimulants for a sustainable agriculture. microorganisms. (2021) 9(2):426. 10.3390/microorganisms9020426 33669534 PMC7922931

[88] Ramstedt M Burmølle M. Can multi-species biofilms defeat antimicrobial surfaces on medical devices? Current Opinion in Biomedical Engineering. (2022) 22:1–8. 10.1016/j.cobme.2022.100370

[89] Tavares LJ Klein MI Panariello BHD Dorigatti de Avila E Pavarina AC. An *in vitro* model of *Fusobacterium nucleatum* and *Porphyromonas gingivalis* in single- and dual-species biofilms. J Periodontal Implant Sci. (2018) 48(1):12–21. 10.5051/jpis.2018.48.1.12 29535887 PMC5841263

[90] O'Hanlon DE Moench TR Cone RA. In vaginal fluid, bacteria associated with bacterial vaginosis can be suppressed with lactic acid but not hydrogen peroxide. BMC Infect Dis. (2011) 11:200. 10.1186/1471-2334-11-200 21771337 PMC3161885

[91] Linhares IM Summers PR Larsen B Giraldo PC Witkin SS. Contemporary perspectives on vaginal pH and lactobacilli. Am J Obstet Gynecol. (2011) 204(2):120e1–5. 10.1016/j.ajog.2010.07.010 20832044

[92] Zhou X Hansmann MA Davis CC Suzuki H Brown C Schutte U The vaginal bacterial communities of Japanese women resemble those of women in other racial groups. FEMS Immunol Med Microbiol. (2010) 58(2):169–81. 10.1111/j.1574-695X.2009.00618.x 19912342 PMC2868947

[93] Forney L Ravel J. National academies of sciences, engineering, and medicine. Community ecology and the vaginal microbiome in microbial ecology in states of health and disease: workshop summary. Washington, DC: The National Academies Press (2014). 292–322. 10.17226/18433 24555208

[94] Boskey ER Telsch KM Whaley KJ Moench TR Cone RA. Acid production by vaginal flora in vitro is consistent with the rate and extent of vaginal acidification. Infect Immun. (1999) 67(10):5170–5. 10.1128/IAI.67.10.5170-5175.1999 10496892 PMC96867

[95] Dufresne K Podskalniy VA Herfst CA Lovell GFM Lee IS DeJong EN Glucose mediates niche-specific repression of *Staphylococcus aureus* toxic shock syndrome toxin-1 through the activity of *ccpA* in the vaginal environment. J Bacteriol. (2022) 204(10):e0026922. 10.1128/jb.00269-22 36106854 PMC9578429

[96] O’Hanlon DE Cone RA Moench TR. Vaginal pH measured in vivo: lactobacilli determine pH and lactic acid concentration. BMC Microbiol. (2019) 19(1):13. 10.1186/s12866-019-1388-8 30642259 PMC6332693

[97] Charlier C Cretenet M Even S LeLoir Y. Interactions between *Staphylococcus aureus* and lactic acid bacterial an old story with new perspectives. Intl J of Food Micro. (2009) 131(1):30–9. 10.1016/j.ijfoodmicro.2008.06.032 18687499

[98] Santos DKF Rufino RD Luna JM Santos VA Sarubbo LA. Biosurfactants: multifunctional biomolecules of the 21st century. Int J Mol. (2016) 17:401. 10.3390/ijms17030401 PMC4813256 26999123

[99] Rodrigues LR Banat IM Van der Mei HC Teixeira JA Oliveira R. Interference in adhesion of bacteria and yeasts isolated from explanted voice prostheses to silicone rubber by rhamnolipid biosurfactant. J Appl Microbiol. (2006) 100(3):470–80. 10.1111/j.1365-2672.2005.02826.x 16478486

[100] Giordani B Costantini PE Fedi S Cappelletti M Abruzzo A. Liposomes containing biosurfactants isolated from *Lactobacillus gasseri* exert antibiofilm activity against methicillin resistant *Staphylococcus aureus* strains. Eur J Pharm Biopharm. (2019) 139:246–52. 10.1016/j.ejpb.2019.04.011 30991089

[101] Morais IMC Cordeiro AL Teixeira GS Nardi RMD Monteiro AS Alves RJ. Biological and physicochemical properties of biosurfactants produced by *Lactobacillus jensenii* P_6A_ *and Lactobacillus gasseri* P_65_. Microb Cell Fact. (2017) 16(1):155. 10.1186/s12934-017-0769-7 28927409 PMC5605992

[102] Velraeds M Van de Belt-Gritter B Van der Mei HC Reid G Busscher HJ. Interference in initial adhesion of uropathogenic bacteria and yeasts to silicone rubber by a *Lactobacillus acidophilus* biosurfactant. J Med Microbiol. (1998) 47(12):1081–5. 10.1099/00222615-47-12-1081 9856644

[103] Meyrand A Vernozy-Rozand C. Growth and enterotoxin production of *Staphylococcus aureus* in different cheeses. Revue Med Vet. (1999) 150:601. 10.1046/j.1365-2672.1998.853531.x

[104] Meyrand A Boutrand Loei S Ray Gueniot S Mazuy C Gaspard CE Jaubert G Growth and enterotoxin production of *Staphylococcus aureus* during the manufacture and ripening of camembert-type cheeses from raw goats’ milk. J Appl Microbiol. (1998) 85(3):537–44. 10.1046/j.1365-2672.1998.853531.x 9750284

[105] Laughton JM Devillard E Heinrichs DE Reid G McCormick JK. Inhibition of expression of a staphylococcal superantigen-like protein by a soluble factor from *Lactobacillus reuteri*. Microbiology. (2006) 152:1155–67. 10.1099/mic.0.28654-0 16549678

[106] Ceresa C Fracchia L Williams M Banat IM Díaz De Rienzo MA. The effect of sophorolipids against microbial biofilms on medical-grade silicone. J Biotechnol. (2020) 309:34–43. 10.1016/j.jbiotec.2019.12.019 31887325

[107] Parsek MR Greenberg E. Sociomicrobiology: the connections between quorum sensing and biofilms. Trends Microbiol. (2005) 13:27–33. 10.1016/j.tim.2004.11.007 15639629

[108] Lewandowski Z Beyenal H Stookey D. Reproducibility of biofilm processes and the meaning of steady state in biofilm reactors. Water Sci Technol. (2004) 49(11-12):359–64. 10.2166/wst.2004.0880 15303762

[109] Arenas-Gallo C Ramírez-Rocha G González-Hakspiel L Merlano-Alcendra C Palomino-Suárez D Rueda-Espinel S. Acceptability and safety of the menstrual cup: a systematic review of the literature. Rev Colomb Obstet Ginecol. (2020) 71(2):163–77. 10.18597/rcog.3425. (in Spanish). 32770872

[110] PATH Menstrual cup cleaning practices: A mixed methods study of published instructions and key informant interviews. Published May (2021). Available at: https://www.path.org/resources/menstrual-cup-cleaning-practices-mixed-methods-study-published-instructions-and-key-informant-interviews/ (Accessed May 15, 2022).

[111] US Centers for Disease Control and Prevention website. (2008) Introduction, methods, definition of terms: Guideline for disinfection and sterilization in healthcare facilities. Available at: https://www.cdc.gov/infectioncontrol/guidelines/disinfection/introduction.html (Accessed May 15, 2022).

[112] Food and Drug Administration. Summary of Safety and Effectiveness Data for PreMarket Approval Application for *FemCap* (Cervical cap). Available at: https://www.accessdata.fda.gov/cdrh_docs/pdf2/P020041b.pdf (Accessed on 3/20/2023).

[113] Rodrigues LR. Inhibition of bacterial adhesion on medical devices. Adv Exp Med Biol. (2011) 715:351–67. 10.1007/978-94-007-0940-9_22 21557075

