# Supplementary material for: Development of in vitro methods to model the impact of vaginal lactobacilli on Staphylococcus aureus biofilm formation on menstrual cups as well as validation of recommended cleaning directions
Source: Front Reprod Health. 2023 Aug 21;5:1162746. doi: 10.3389/frph.2023.1162746 (PMC10475951; doi:10.3389/frph.2023.1162746)
Supplement: Supplementary file 1 [file Datasheet1.docx]

***Supplemental Information***

**Development of in vitro Methods to Model the Impact of Vaginal Lactobacilli on *Staphylococcus* *aureus* Biofilm Formation on Menstrual Cups as Well as Validation of Recommended Cleaning Directions**

Maria Friberg, Kara Woeller*, Vighter Iberi, Paolo Palacio Mancheno, James Riedeman, Lisa Bohman, and Catherine C. Davis

***Corresponding Author**

**woeller.ke@pg.com**

**Table S-1: Tampax Menstrual Cup Identification Information by Study**

| **Study** | **Material Name** | **Identification #** | **Material Type** |
| --- | --- | --- | --- |
| Void Volume Estimates | QP1-40 | 91919500 TMCH37 | Class VI Medical Grade Silicone |
| Magnetic Resonance Imaging | QP1-40 | EXP-17-BS3644 9151017 | Class VI Medical Grade Silicone |
| Biofilm Quantitative Measurement | QP1-40  QP1-20, (Prototype 1)  QP1-20. (Prototype 2) | EXP-21-EK0739 PE: 11882  EXP-21-EK0739 PE:1181  EXP-21-EK0739 PE:1181 | Class VI Medical Grade Silicone  Class VI Medical Grade Silicone  Class VI Medical Grade Silicone |
| Qualitative/visual measurement via uCT and Cryo SEM  *Staphylococcus aureus* only  *Staphylococcus aureus* and *Lactobacillus gasseri* | QP1-40  QP1-20 (Prototype 1)  QP1-20 (Prototype 2) | EXP-21-EK0739 PE: 11882  EXP-21-EK0739 PE:1181  EXP-21-EK0739 PE:1181 | Class VI Medical Grade Silicone  Class VI Medical Grade Silicone  Class VI Medical Grade Silicone |
| Cleaning Effectiveness | QP1-40 | 91970718 TMCH37 | Class VI Medical Grade Silicone |
| ***Legend Table S-3:*** *The currently marketed Tampax menstrual cup is comprised of QP1-40 Class VI medical grade silicones supplied by Dow/Dupont. QPI-20 menstrual cup is not marketed but is identical to the QP1-40 menstrual cup with the exception of minor differences in starting materials. The difference between Prototype 1 and Prototype 2 is the initiation of a 4 hour curing step which was utilized for Prototype 1 only. All testing was conducted on Tampax menstrual cups, size “heavy flow”.* | | | |

**Table S-2. Inclusion and Exclusion Criteria**

| **Inclusion Criteria** | **Exclusions Criteria:** Subjects will be excluded if one or more of the following apply: |
| --- | --- |
| 1. be a currently active female research panelist registered with the Research Center and have a current completed annual Medical Screening Questionnaire on file at that facility which has been approved by the facility’s consulting gynecologist 2. be at least 18 years of age 3. have signed the informed consent form (ICF) for this trial 4. have agreed to abide by the trial requirements and restrictions 5. have completed a research panelist self assessment (Attachment I) 6. be in good gynecological health per the medical screening 7. be in good general health (self reported) 8. have a recurring menstrual cycle (for tampon studies only) 9. occasionally use tampons (for tampon studies only) 10. have refrained from vaginal sexual intercourse, using douches, or vaginal creams for 48 hours when requested for specific trials 11. be willing to lie in a MRI (enclosed and/or open) 12. be willing to remove jewelry and under wire bra or clothing and accessories with metal components during MRI   be willing to comply with the MRI research facility’s requirements | 1. self reports being pregnant or if subject unsure, positive results with pregnancy test. A pregnancy test is automatically conducted if the menstrual cycle extends longer than normal for the subject. 2. self reports having diabetes 3. self reports a history of Toxic Shock Syndrome (tampon studies only) 4. has difficulty wearing tampons or have had irritation from the use of a tampon product (tampon studies only) 5. has known latex allergies or sensitivity to tape or adhesive 6. self reports having an active vaginal infection (yeast, bacterial) or Urinary Tract Infection (UTI) with symptoms or currently being treated for a vaginal infection or UTI 7. experiencing a change in vaginal odor, dryness, itching, burning sensations, abnormal discharge or non-menstrual vaginal bleeding (Research Panelist Self Assessment Form) 8. experiencing open sores, rashes, or lesions in the genital area (Research Panelist Self-Assessment Form) 9. does not meet the requirements of the MRI facility 10. has cochlear implants 11. has orthopedic hardware 12. has hearing aides 13. has an insulin/infusaid pump 14. has a cardiac pacemaker, heart valves or lead wires 15. has brain aneurysm clips 16. has interuterine devices 17. has implanted tens unit or lead wires 18. has implanted bone stimulator or lead wires 19. has metal fragment in the eye or shrapnel near heart or spinal cord |

**Table S-3: *S. aureus* Growth Mode Studies with Feminine Hygiene Products*:* Comparison of Study Methodology as Published by Reiser et al, Schlievert et al, Nonfoux et al, and Friberg and Woeller et al.**

| **Element** | **Reiser et al.** | **Nonfoux et al.** | **Schlievert** | **Friberg and Woeller et al.** |
| --- | --- | --- | --- | --- |
| Test method | Tampon sac | Tampon sac with major deletions of Reiser protocol | Stationary flask, shake flask, tampon sac (Reiser) with minor modifications | Mixed microflora Assay stationary flask with minor modifications |
| Test products | Tampons | Tampons, diaphragms, and menstrual cups | Tampons, diaphragms, and menstrual cups | Menstrual cups |
| Test design | Quantification of *S. aureus* and TSST-1 | Quantification of *S. aureus* and TSST-1 (planktonic and biofilm) and characterization of *S. aureus* biofilm formation | Quantification of *S. aureus* and TSST-1 | Quantification of *S. aureus* (planktonic and biofilm) and characterization of *S. aureus* biofilms |
| Organism | *S. aureus* FRI-1169 (high producer), FRI-1183 and FRI-1187 (low producer) | *S. aureus* ST20140321 | *S. aureus MN8*  Hyper-producer TSST-1; 8^th^ | *S.aureus* ATCC 33589  *S. aureus subsp aureus* Rosenbach D1470 (587) |
| Organism source | Isolated from TSS patient | Isolated from tampon of menstrual TSS patient | Isolated from TSS in Minnesota – not specified if from mTSS patient | Isolated from vagina of TSS case |
| Organism genetics | *tst* inserted in tyrosine operon | *Agr-3,* methicillin-sensitive, clonal complex 30, TSST-1 producing strain | USA200 Clonal Complex 30/30 ST36 clonal group; common mTSS spa type | TSST-1 producer |
| **Quantitative Assessment** | | | | |
| Test sample prep | Intact tampon | Intact cups | Intact tampons and cups tested as 2.5 cm pieces | Intact cup |
| Inoculum concentration of Organism(s) | 10^5^ CFU/mL | 10^5^ CFU/mL | Flasks: 5.8 x 10^6^ CFU/ml  Tampon sac: 6.3 x 10^6^ CFU/mL | *L. gasseri:*10^8^ CFU/mL  *S. aureus:*10^4^ CFU/mL |
| Final concentration of Organism(s) | 5.8 X 10^10^ + 2.6 x 10^10^ CFU/ml | 2.5 x 10 ^9^ + 2.0 x 10^8^ CFU/ml | Stationary flask: ~1 x 10^10^ CFU/mL  Shake flask: ~1 x 10^10^ CFU/mL  Tampon Sac: ~1 x 10^10^ CFU/mL | *L. gasseri:* 10^8^ CFU/mL  *S. aureus:* 10^6^ CFU/mL |
| Media and Volume for incubation | 100 mL of BHI  Agar surrounds cellulose tampon sac containing broth & inoculum – oxygen available only from entrapped air in tampons | 15 mL of BHI broth “into cavity of the cup”  Cup placed in Whirlpak bags and air manually expressed, bag hermetically sealed | 10 ml of THB | 250mL VDM in beaker |
| Incubation parameters | 18 hrs @37^0^C static  50°C at initiation and allowed to cool to 37°C | 8 hrs @37^0^C static | Flask: 12 hrs @37^0^C with and without shaking @ 200 RPM  Tampon sac: 12 hrs static; assume @ 37°C | 12 hrs @35+2^0^C static |
| Test conditions | Anaerobic | Aerobic | Aerobic (flask); Anaerobic (tampon sac) | Anaerobic |
| Cleaning procedure | NA | Cleaned 3x with 300 mL of PBS | Not specified | Cleaned 3x with 300 mL PBS |
| Organism recovery method | Placed in whirl pack bag containing sterile buffered saline with Tween (0.01 M sodium phosphate, 0.9% NaCl, pH 7.5. 0.05% Tween20) added at 4x the weight of the tampon, tampon stomached inside whirlpak bag | Sonication in 150 mL PBS for 10 minutes | Not specified | Sonicated in 200 mL of PBS for 1 minute |
| *S. aureus (*and *L.gasseri)* quantification | Total *S. aureus* CFU quantified by plating serial dilutions of the extract on plate count agar; type of agar not specified | Flow cytometry and recovery on TSA plate and counting of *S. aureus* with Scan 1200 colony counter after 24 hr incubation at 37°C | Not specified | Organism plated [MSA for *S. aureus*  (aerobic) and HBT for *L. gasseri* (anaerobic)*,* incubated at 37°C and hand counted after 24 and 48hr (*S. aureus)* and 48 hours (*L. gasseri)]* |
| Statistical analysis | Not specified | *t* test | *t* test | ANOVA |
| **Qualitative Assessment** | | | | |
| Test sample | NA | 1 x 1.5 cm sections | 2.5 cm pieces | Intact cup |
| Organism | NA | *S. aureus* LUG2902  Utilized pACL1484 plasmid to optimize maximal fluorescence | NA | *S.aureus* ATCC 33589  *S. aureus subsp aureus* Rosenbach  D1470 (587) isolated from vagina of TSS case |
| Inoculum concentration of organism | NA | 10^7^ CFU/mL in 24 well plate Falcon plate | NA | *L. gasseri:*10^8^ CFU/mL  *S. aureus:*10^4^ CFU/mL |
| Final concentration of Organism(s) | NA | Not specified | NA | *L. gasseri:* 10^8^ CFU/mL  *S. aureus:* 10^6^ CFU/mL |
| Media and Volume for incubation | NA | 3 mL of BHI broth | NA | 250mL VDM in beaker |
| Incubation parameters | NA | 2, 4, 6, and 8 hours at 37°C static | NA | 12 hrs @35+2^0^C static |
| Cleaning procedure | NA | 5 times with 2 mL PBS | NA | NA – placed on dry ice, stained with osmium tetroxide and evaluated |
| Biofilm visualization method | NA | Confocal microscope  GFP-labeled *S. aureus* | NA | uCT used to ID area; cyro-SEM used to visualize organisms/biofilm |
| ***Legend Table S-1:*** *NA: Not applicable; BHI: Brain Heart Infusion; THB: Todd Hewitt Broth; VDM: Vaginal Defined Media; ANOVA: Analysis of Variance; PBS: Phosphate Buffered Saline; MSA: Mannitol Salt Agar; HBT: Human Blood Tween; uCT: Micro-computed tomography; cyro-SEM: cyro Scanning Electron Microscopy* | | | | |

**Table S-4: Menstrual Cup Cleaning Studies - Comparison of Study Methodology as Published by Wunsch et al and Friberg and Woeller et al**

| **Element** | **Wunsch et al Published method** | **Friberg & Woeller et al** |
| --- | --- | --- |
| Soil | Human blood from lab worker | nAMF |
| Organism | *S. aureus* DSM 25630 | *S. aureus* ATCC 33589  *E. coli* ATCC 8739 |
| Application of Soil to Menstrual cup | Soil applied to cup, allowed to air dried, placed in beaker with s. aureus, allowed to sit overnight | Soil with organism applied to menstrual cup and allowed to air dry for ~1 hour before cleaning study |
| Organism genetics | TSST-1 producer  MRSA | *S. aureus: S. aureus subsp aureus* Rosenbach  D1470 (587); isolated from vagina of TSS case  *E. coli:* Migula Castellani and Chalmers; isolated from feces |
| Inoculum concentration of organism | Final conc in beaker 10^6^CFU/mL | Final conc applied directly to the cup: 5x10^7^ cfu total on cup  (10^5^ cfu/mL after sonication in 200 mL PBS) |
| Media and Volume for incubation | 125mL TSB | No incubation step. Organisms are applied directly to the cup in a 1:1 mix of nAMF and allowed to dry at ambient temperatures for 1 hr |
| Media and Volume for organism retrieval | 10 mL sterile NaCl-Tween | 200ml 1x PBS |
| Organism recovery method | stomaching | 5 min sonication |
| Agar plates | TSA | MLAT |
| Incubation parameters of agar plates after test | 12hrs @37+1^0^C | 48hrs @35+2^0^C |
| Results | Positive Control: 2.075x10^8^CFU/cup  Clean w/soap 30s: 7.9x10^3^CFU/cup  >4Log 99.996% | Positive Control (*S.aureus, no cleaning)*: 4x10^7^CFU/cup (average)  Clean w/soap 30s: 1.7 x10^4^CFU/cup  >3Log 99.9% |
| ***Legend Table S-2:*** *nAMF = New Artificial Menstrual Fluid; MRSA = Methicillin-Resistant Staphylococcus aureus; TSA: Tryptic Soy Agar; TSB: Tryptic Soy Broth; MLAT: Modified Letheen Agar with Tween* | | |

**References for Supplement:**

1. Reiser RF, Hinzman SJ, Bergdoll MS. Production of Toxic Shock syndrome Toxin 1 by *Staphylococcus aureus* Restricted to Endogenous Air in Tampons. J. Clin Micro. 25(8): 1450-1452. 1987. doi: [10.1128/jcm.25.8.1450-1452.1987](https://doi.org/10.1128%2Fjcm.25.8.1450-1452.1987)
2. Nonfoux L, Chiaruzzi M, Badiou C, Baude J, Tristan A, Thioulouse J, Muller D, Prigent-Combaret C, Lina G. Impact of Currently Marketed Tampons and Menstrual Cups on *Staphylococcus aureus* Growth and Toxic Shock Syndrome Toxin Product *In Vitro.* Applied and Environmental Microbiology. 84(12): 1-12. 2018. <https://doi.org/10.1128/AEM.00351-18>.
3. Schlievert PM. Effect of Non-Absorbent Intravaginal Menstrual/Contraceptive Products on *Staphylococcus aureus* and Production of the Superantigen TSST-1. European Journal of Clinical Microbiology & Infectious Diseases. 39:31-38 2020 <https://doi.org/10.1007/s10096-019-03685-x>
4. Wunsch N, Green SJ, Adam S, Hampton J, Phillips-Howard PA. Mehta SD, *In Vitro* Study to Assess Effective Cleaning Techniques for Removing *Staphylococcus aureus* from Menstrual Cups. International Journal of Environmental Research and Public Health. 19, 1450: 1-11. 2022. doi.org/10.3390/ijerph19031450
